# The adult boar testicular and epididymal transcriptomes

**DOI:** 10.1186/1471-2164-10-369

**Published:** 2009-08-07

**Authors:** Benoît Guyonnet, Guillemette Marot, Jean-Louis Dacheux, Marie-José Mercat, Sandrine Schwob, Florence Jaffrézic, Jean-Luc Gatti

**Affiliations:** 1 UMR85 Physiologie de la Reproduction et des Comportements, Institut National de la Recherche Agronomique, F-37380 Nouzilly, France; 2 UMR6175 Physiologie de la Reproduction et des Comportements, Centre National de la Recherche Scientifique, F-37380 Nouzilly, France; 3 Université François Rabelais de Tours, F-37041 Tours, France; 4 Haras Nationaux, F-37380 Nouzilly, France; 5 Pôle Génétique, IFIP Institut du Porc, F-35650 Le Rheu, France; 6 UR337 Station de Génétique Quantitative et Appliquée, Institut National de la Recherche Agronomique, F-78350 Jouy en Josas, France

## Abstract

**Background:**

Mammalians gamete production takes place in the testis but when they exit this organ, although spermatozoa have acquired a specialized and distinct morphology, they are immotile and infertile. It is only after their travel in the epididymis that sperm gain their motility and fertility. Epididymis is a crescent shaped organ adjacent to the testis that can be divided in three gross morphological regions, head (caput), body (corpus) and tail (cauda). It contains a long and unique convoluted tubule connected to the testis via the efferent ducts and finished by joining the *vas deferens *in its caudal part.

**Results:**

In this study, the testis, the efferent ducts (*vas efferens*, VE), nine distinct successive epididymal segments and the deferent duct (*vas deferens*, VD) of four adult boars of known fertility were isolated and their mRNA extracted. The gene expression of each of these samples was analyzed using a pig generic 9 K nylon microarray (AGENAE program; GEO accession number: GPL3729) spotted with 8931 clones derived from normalized cDNA banks from different pig tissues including testis and epididymis. Differentially expressed transcripts were obtained with moderated t-tests and F-tests and two data clustering algorithms based either on partitioning around medoid (top down PAM) or hierarchical clustering (bottom up HCL) were combined for class discovery and gene expression analysis. Tissue clustering defined seven transcriptomic units: testis, *vas efferens *and five epididymal transcriptomic units. Meanwhile transcripts formed only four clusters related to the tissues. We have then used a specific statistical method to sort out genes specifically over-expressed (markers) in testis, VE or in each of the five transcriptomic units of the epididymis (including VD). The specific regional expression of some of these genes was further validated by PCR and Q-PCR. We also searched for specific pathways and functions using available gene ontology information.

**Conclusion:**

This study described for the first time the complete transcriptomes of the testis, the epididymis, the *vas efferens *and the *vas deferens *on the same species. It described new genes or genes not yet reported over-expressed in these boar tissues, as well as new control mechanisms. It emphasizes and fulfilled the gap between studies done in rodents and human, and provides tools that will be useful for further studies on the biochemical processes responsible for the formation and maintain of the epididymal regionalization and the development of a fertile spermatozoa.

## Background

Spermatozoa are produced in the testis as the result of a complex assembly line that makes a highly shaped cell, morphologically and biochemically specialized. These cells are released in the seminiferous tubules fluid and then begin a long journey by "floating" passively through the rete testis to leave the testis, entering the efferent ducts and thereafter moving to the *unique *epididymal duct. Testicular spermatozoa are non functional, it is solely before reaching the end of the epididymis, (where they will be stored from days to weeks upon the species) that they will acquire their physiological functions: the possibility of motility, of undergoing the post-ejaculatory events (ie, capacitation and hyperactivation), the ability to recognize and to bind to the oocyte investments and plasma membrane, and in short their natural fertilizing ability [1,2].

This post-testicular maturation results from changes in the sperm membrane composition and metabolism in response to the profound alterations of the surrounding fluid composition. The fluid components (ions, chemicals, lipids and proteins) are changing all along the different ducts through which the sperm transit [2-4]. It is now well established that the sperm changes are progressive and occur in a define order. Among the main transformations, the changes in the sperm surface proteins have been extensively described [2,5-7]. A number of sperm surface components are removed or altered during the transit while others are added in relation with the fluid changes. Some of these surface component changes have been involved in the sperm egg recognition but these components and therefore their mechanisms of modification are not "universal" since they are not found in all mammals (i.e. Fertilin-alpha which is involved in mouse sperm-egg recognition but is a pseudogene in human) [8-11]. Moreover mouse knockout approaches to prove their role in fertilization has been found tedious since the absence of one component during spermatogenesis or sperm epididymal maturation may result in improper formation of specialized sperm membrane surface domains [9,12,13]. These strongly suggest that multispecies studies are necessary to better understand the common as well as species-specific mechanisms involved in the sperm maturation process and fertility.

The epididymal secretome and proteome of several mammalian species including pigs as well some of the mechanisms involved in the sperm surface alteration have been recently described [14-18]. The complete sequencing of several mammalian genomes and the possibility open by technologic progresses allow now analyzing the complete gene expression of a tissue by microarray. Nowadays more than ten different transcriptomic studies of mouse, rat and human epididymis have been achieved [19-25]. These studies have shown that a large number of genes are expressed in the epididymis but only few genes were found specifically over-expressed in this organ compared to other tissues. Meanwhile they have also showed that a high level of regionalization of the expression of part of these genes existed and that this gene expression (and hence protein secretions) is tightly regulated by the hormonal status and also by very specific local mechanisms present either in the fluid within the tubule and/or may be by action from the embedded fluid surrounding the tubule in a specified zone delimited by the connective *septae *of the supporting tissue [23,25]. However, none of these studies have included a comparison with testicular gene expression and with the *vas efferens *and *vas deferens *transcriptomes and the human studies have been limited to the three gross morphological parts of the epididymis (caput, corpus, cauda). Here we have used a microarray approach to analyze the transcriptome of nine defined regions of adult fertile boar epididymis, efferent and deferent ducts and testis. These epididymal regions were chosen according to previous studies done on this species [1,26].

Pigs provide good model for biomedical research, as pigs and humans show in many aspects physiological, biochemical, pathological and pharmacological similarities. Recently it has been demonstrated that the porcine and the human genome evolutionary distance is smaller than the distance between mouse and human [27]. For example a survey using a pig 20 000 genes cDNA microarray indicated that gene expression in common porcine tissues is very similar to the expression in homologous tissues of human [28]. This provides a rationale to use porcine sequences in gene expression comparisons with human in transcriptomic analysis. Moreover, pig tissues are more easily available than their human counterparts and several pig microarrays have been made available to screen for genes involved in specific biological processes of interest such as diseases or development, but also related to the agronomical interest for this species [29] since pigs are one of the most eaten animals in the world today and the pig industry will use more and more genetic selection based on the development of marker-assisted selection that rely on the mastering of artificial insemination techniques, including the sperm fertility.

This study then fulfill the gap between the rodent and human by addressing a domestic species with an agronomical interest where the epididymal duct measure more than 50 meters long and the transit of the sperm is at least three weeks.

We have used a microarray that was made from clones issued from a multi-tissue cDNA normalized library including the testis and the three main gross regions (caput, corpus, cauda) of the epididymis [30]. We have also adapted specific statistical methods to analyze the gene clustering and procedure to obtain genes that were over-expressed in specific transcriptomic units. Our data showed the expression of several new genes in the epididymis and confirmed that very few epididymal specific genes existed. Meanwhile a certain number of genes have a very tight expression in one or a few zones and then could be useful as markers for the epididymal activity and some of these genes will also certainly be good candidates to further understand the very specific biochemical processes involved in epididymal regionalization and to obtain fertile spermatozoa.

## Results

### Differentially expressed transcripts between 12 conditions

**Figure 1:**
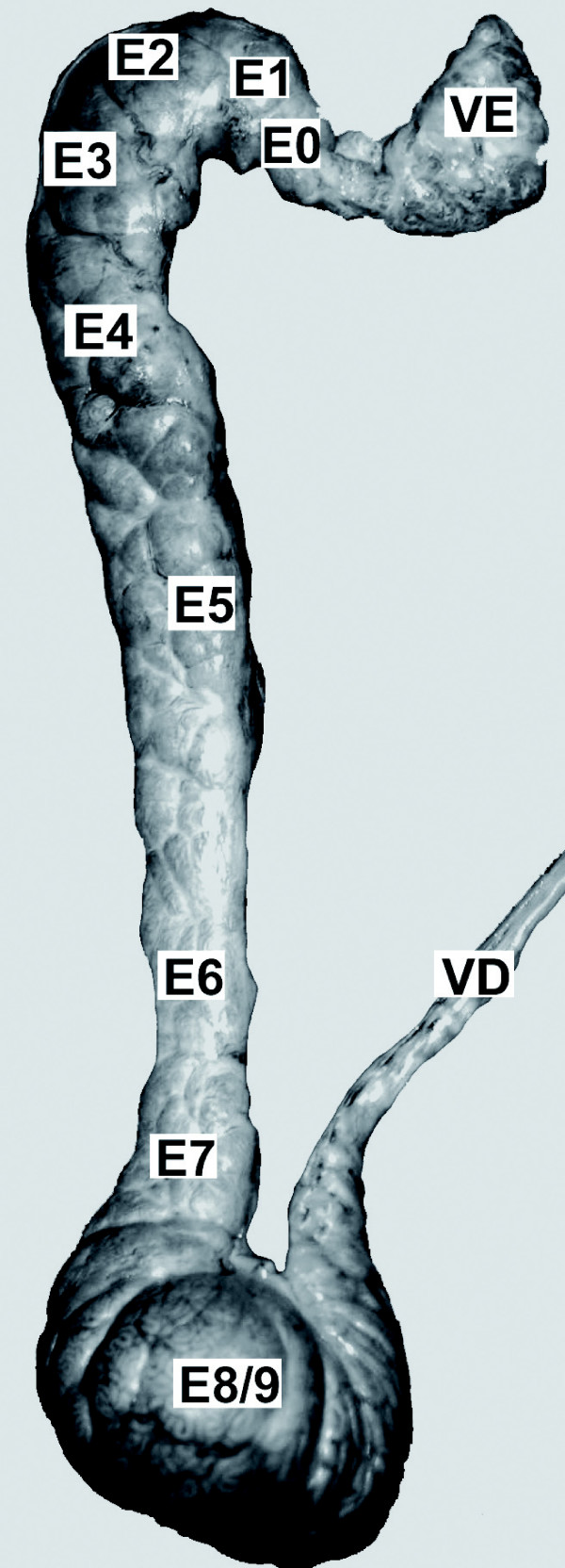
**Boar epididymis**. Picture of the boar epididymis showing the different regions sampled: VE, *vas efferens*; 0 to 8/9, epididymis; VD, *vas deferens*. Reel size, 30 cm.

The extracted mRNAs from the 12 different samples (testis, *vas efferens*, epididymis (zones 0 to 8/9) and *vas deferens *(Figure 1)) were retro-transcribed in presence of ^33^P-labelled oligonucleotide and hybridized onto the Nylon microarray spotted with PCR products from clones from a multi-tissues bank including reproductive tissues from male and female [30]. Each PCR product contained also a short sequence used to estimate the quality of the membrane and to measure the quantity of each clone spotted. This last value was used to correct the signal after hybridization with the ^33^P-cDNA.

After cleaning and digital processing of our array data, for each tissue sample only spots with at least 3 valid replicates were kept to estimate the intra-group variability. We then retained 7676 valid spots throughout the 48 membranes and 12 samples studied.

In order to determine the differential expression of a transcript between the twelve different tissue samples, each was compared to the other for these 7676 spots with a moderated F-test from the Limma R package (p-value threshold after BH correction was 0.00001). Between the 12 tissue samples, 2115 transcripts showed a differential expression. At this step, we expected that the main housekeeping genes or the genes that were expressed at the same level in all the tissues tested have been removed from the analysis.

### Transcriptional units

**Figure 2:**
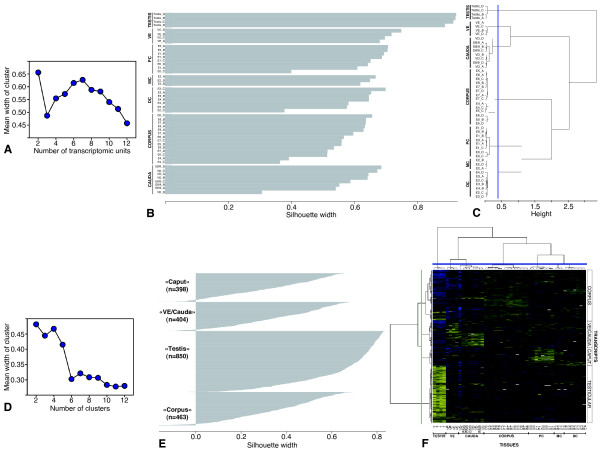
**Statistical analysis**. **A) **PAM outcome: the graph shows the mean width of clusters depending on the number of zones included in the cluster. **B) **Silhouette width (clustering score) of each of the seven transcriptomic units chosen for the clustering using the best second score of the PAM algorithm. **C) **Dendrogram obtained for the clustering of the 12 zones by HAC algorithm. The blue line cuts the dendrogram to retrieve the 7 transcriptomic units. **D) **Graph of PAM outcome showing the mean width of clusters depending on the number of clusters for transcripts. **E) **Clustering score (silhouette width) obtained for transcripts corresponding to the second best score of the PAM. **F) **Heat map representation of the transcripts clustering obtained by HCL algorithm. The blue line at the top cuts the dendrogram to show the 7 transcriptomic units and the green line on the left cuts the dendrogram to retrieve the 4 clusters of transcripts. (Relative expression level: yellow = high, blue = low; white = missing values). T: Testis, VE: *Vas efferens*, E0 to E8/9: nine epididymal zones, PC: Proximal caput, MC: Median caput, DC: Distal caput, VD: *Vas deferens*.

We first analyzed how these 2115 differentially expressed transcripts could classify our biological samples. A clustering of the 12 tissue conditions was then performed with the combinative use of the PAM and the HAC. The PAM gives a graphic profile to help in the choice of the number of classes, which is a critical step in unsupervised methods (Figure 2A). In this figure, the quality of the classification (measured by the mean silhouette width) is dependent on the number of classes chosen. The higher is the value of the mean silhouette width; the better is the quality of classification. In our case as it could be expected, the best fit was for two classes (testis versus the other tissues), but this was poorly informative from a physiological point of view. We then decided to retain the second best score with 7 classes. Figure 2B shows how the arrays are clustered within these 7 classes with the score obtained for each array.

The result obtained by the HAC analysis is shown in Figure 2C. It was possible to find a threshold on the dendrogram to retrieve the 7 clusters grouping the same tissue arrays as the PAM. By combining the results of these two methods, the 7 clusters that fit the best our data of gene expression correspond to: (i) the testis, (ii) the efferent ducts, (iii) the anterior caput of the epididymis (zones 0–1), (iv) the median caput (zone 2), (v) the posterior caput (zones 3–4), (vi) the corpus (zones 4–7) and (vii) the caudal region with the zone 8/9 and the deferent duct. We also observed that some uncertainties exist for some arrays, certainly due to animal variations: one epididymal zone 2 membrane was classified in the posterior caput while some zone 4 membranes were clustered within the corpus unit instead in the posterior caput as expected.

### Co-expressed transcripts

With the combinative use of the PAM and the HCL, we analyzed how many co-expression classes could be formed by the 2115 differentially expressed transcripts (Figure 2). With the PAM the best classification of co-expressed transcripts gave again two main clusters: the testicular transcripts versus the other tissues transcripts (Figure 2D). The classification with the second best score gave 4 clusters. As shown in Figure 2E, for each transcript a value was associated which represents how the transcript fits in the classes, the higher is the value the better this transcript is representative of this class. The HCL analysis showed also two main branches (testicular transcripts *versus *the other tissues transcripts) and we were able with a threshold to separate 4 clusters identical to the PAM (Figure 2F). Then the analysis of the transcripts co-expression defined four main classes: (i) the testis, (ii) the head of the epididymis, (iii) the corpus and (iv) the efferent ducts and the caudal region (E8/9 and deferent duct). The heat map from the HCL (Figure 2F) showed the intensity of each co-expressed transcripts within a cluster; some of them had only a very small differential expression or could have biphasic expression.

From these results, the identifier of each transcript from each cluster were used to build a contingency table to establish a list of robust transcripts classified the same way by the two different algorithms (Table 1). Among the 2115 differentially expressed transcripts and the 12 tissue conditions, 1772 (83%) are clustered in the same way by the two statistical methods used. Among them, 774 (36%) transcripts have a preferential expression in the testis, 226 (11%) in the head of the epididymis, 453 (21%) in the corpus and 319 (15%) in the efferent ducts and the caudal region (zone 8/9 and deferent duct). 343 (17%) transcripts were not classified the same way by the two methods.

**Table 1:** Search for common co-expressed transcripts

Cluster	HCL	PAM	Common transcripts
Testis	794	850	774
Caput	250	398	226
Corpus	702	463	453
VE/Cauda	369	404	319

Common transcripts are those clustered in the same way by the HCL and PAM algorithms.

### Search for preferentially over-expressed transcripts between the 7 transcriptomic units

**Figure 3:**
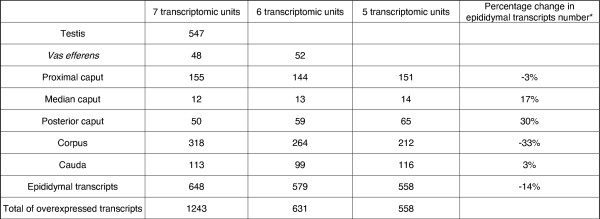
**Over-expressed transcripts**. Number of over-expressed transcripts in an unique transcriptomic unit for each of the 7 transcriptomic units found by the clustering analysis (BH = 1/100 000). Analysis was done with all transcriptomic units (7 units), without testis (6 units) and without testis and *vas efferens *(5 epididymal units). *compared results obtained with 5 *versus *7 transcriptomic units.

The clustering analysis has shown that our 12 tissue conditions formed 7 informative transcriptomic units. Meanwhile the gene analysis showed only four classes of co-expressed genes. We then decided to dig further our data to determine which transcripts are preferentially over-expressed in only one of the transcriptional units. To do this, we used parwise moderated t-tests from the SMVar package [31] (for more detailed explanations see materials and methods). We first searched iteratively for transcripts differentially expressed between 2 transcriptomic units with the pairwise moderated t-test, then for each unit, we selected over-expressed transcripts using the value of the logRatio expression obtain during the test. Then, we compared between units, the list of over-expressed transcripts and retained only those belonging to one single list. Doing this, we selected, for each transcriptomic unit, transcripts that are preferentially over-expressed compared to the others. This was done on the 7676 usable spots and the 7 informative transcriptomic units. The results of this test are summarized in Figure 3: 1243 unique transcripts have a preferential over-expression in one of these units.

Meanwhile, because we knew that some genes could be expressed both in the testis and also in the epididymis (ie, clusterin, PTGDS [5,32,33]), these genes may be eliminated by our analysis although they are over-expressed in one unit of the epididymis. The analysis was then redone in excluding the testis: we then found that 631 transcripts were over-expressed in only one of the 6 remaining units. We have also done the same approach excluding both the testis and the *vas efferens *that may have a partially different embryonic origin than the epididymis [34,35], and found 558 transcripts over-expressed in one of the epididymal units.

Because including the testis and the *vas efferens *in our analysis had consequence on numbers of transcripts retrieved as epididymal, we performed a second analysis on the 7676 qualified spots without these tissues. The Limma moderated F-test (BH p-value threshold of 0.00001) gave 850 transcripts differentially expressed between the nine epididymal zones and the *vas deferens*. When we compared this new list with the list of differentially expressed transcripts between the 12 conditions, we observed that 838 transcripts were common and only 12 were new ones. The classification on the nine epididymal zones and the *vas deferens *was performed with 2 algorithms (PAM and the HAC) gave 5 tissue clusters corresponding to (i) the anterior caput of the epididymis (zones 0–1), (ii) the median caput (zone 2), (iii) the posterior caput (zones 3–4), (iv) the corpus (zones 4–7) and (v) the caudal region (zone 8/9) including the *vas deferens*. A clustering of co-expressed transcripts with the combinative use of the PAM and the HCL on the 850 differentially expressed transcripts found only 3 clusters: (i) the head of the epididymis, (ii) the corpus, (iii) the caudal region.

A contingency analysis showed that 721 transcripts among the 850 are clustered in the same way by the 2 statistical approaches used. Among them, 220 have a preferential expression in the head of the epididymis, 343 in the corpus and 158 in the caudal region (zone 8/9 and *vas deferens*).

### Search for potential markers (testis, *vas efferens*, epididymis)

Numerous genes were found over-expressed in more than one tissue. As we were interested to find more specific genes and functions related to these genes, we decided to search for "markers". Markers are then defined as transcripts that are over-expressed preferentially in the same transcriptomic unit where the clustering analysis classified them. By this way, we do not keep as markers transcripts that have a biphasic expression profile. By crossing the data, for the testis 459 potential markers were found, for the efferent ducts 33, for the anterior head 116 (zones E0-1), for the median head (zone E2) 8 potential markers, for the posterior head (zones E2-3-4) 14, for the corpus (zones E4-5-6-7) 90 and for the cauda (zone E8/9 and deferent duct) 43 transcripts (See List in Additional File 1).

### Validation of microarray by semiquantitative RT-PCR and real-time PCR

We have selected 20 different transcripts found as differentially expressed by the clustering analysis and then designed primers (Additional File 2) and measured the relative amplicon intensities obtained after 25, 30, 35 and 40 cycles of RT-PCR amplification.

**Figure 4:**
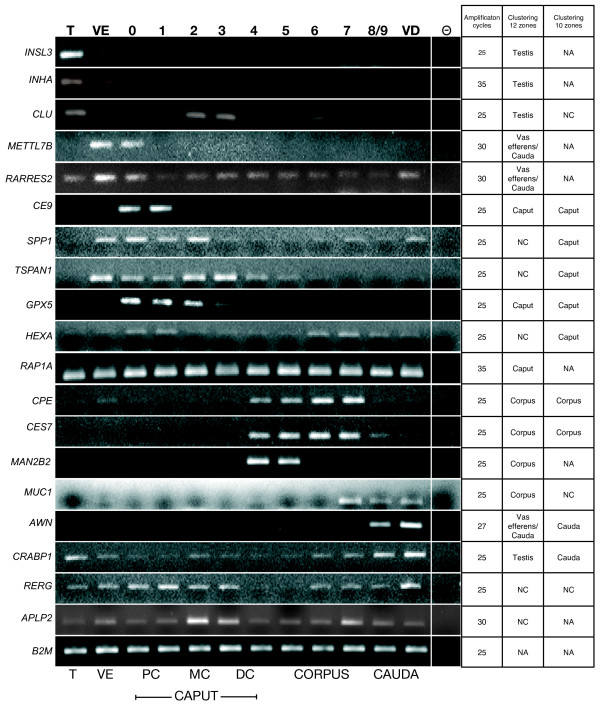
**RT-PCR amplifications**. RT-PCR amplifications of some transcripts differentially expressed or control (ie, not differential) between the 12 or 10 zones studied. T: Testis, VE: *Vas efferens*, PC: Proximal caput (E0-1), MC: Median caput (E2), DC: Distal caput (E3-4), Corpus (E4-7), Cauda (E8/9 and *Vas deferens *(VD)), (-): negative PCR controls. The table on right indicated the number of PCR cycles used and the cluster in which the transcript belongs by the clustering analysis using 12 or 10 zones. NC: transcripts were not classified in an identical manner by the two methods (PAM and HCL), NA: transcripts were not statistically differentially expressed. *INSL3 *(insulin-like 3), *INHA *(inhibin, alpha), *CLU *(clusterin), *METTL7B *(methyltransferase like 7B), *RARRES2 *(retinoic acid receptor responder (tazarotene induced) 2), *TEDDM1 *(Transmembrane epididymal protein 1), *SPP1 *(secreted phosphoprotein 1), *TSPAN1 *(tetraspanin 1), *GPX5 *(glutathione peroxidase 5 (epididymal androgen-related protein)), *HEXA *(hexosaminidase A (alpha polypeptide)), *RAP1A *(RAP1A, member of RAS oncogene family), *CPE *(carboxypeptidase E), *CES7 *(carboxylesterase 7), *MAN2B2 *(mannosidase, alpha, class 2B, member 2), *MUC1 *(mucin 1, cell surface associated), *AWN *(sperm associated AWN protein), *CRABP1 *(cellular retinoic acid binding protein 1), *RERG *(RAS-like, estrogen-regulated, growth inhibitor), *APLP2 *(amyloid beta (A4) precursor-like protein 2), *B2M *(beta-2-microglobulin).

Almost all transcripts were found effectively expressed within the transcriptomic unit assigned by the statistical approach (Figure 4). Only for RAP1A, which had a very low expression level on microarray, it was not possible to observe the differential expression by RT-PCR. This figure also confirms that some of the genes have a bi-phasic expression: they were both expressed in the testis and in one transcriptomic unit of the epididymis. These genes could then be found over-expressed in one unit when the statistical analysis was done on the twelve conditions but like *CRABP1 *or *AWN*, become part of an epididymal cluster when the analysis was done without the testis and the *vas efferens *(10 tissue conditions). As control we also amplified two genes (*RERG *and *APLP2*) that were not found in the common list of the two statistical approaches but had a statistically significant differential expression on microarray. Again the results of microarray and RT-PCR were in agreements.

We also analyzed the beta 2-microglubulin (*B2M*) gene that was not found differentially expressed. The primers for this gene were designed in such way that they were on two successive exons. The RT-PCR showed only the waited amplicon of 189 bp (instead of 757 bp when the intron is included) indicating also the absence of genomic DNA contamination. As predicted this gene showed no differential expression by PCR whatever the number of PCR cycles.

**Figure 5:**
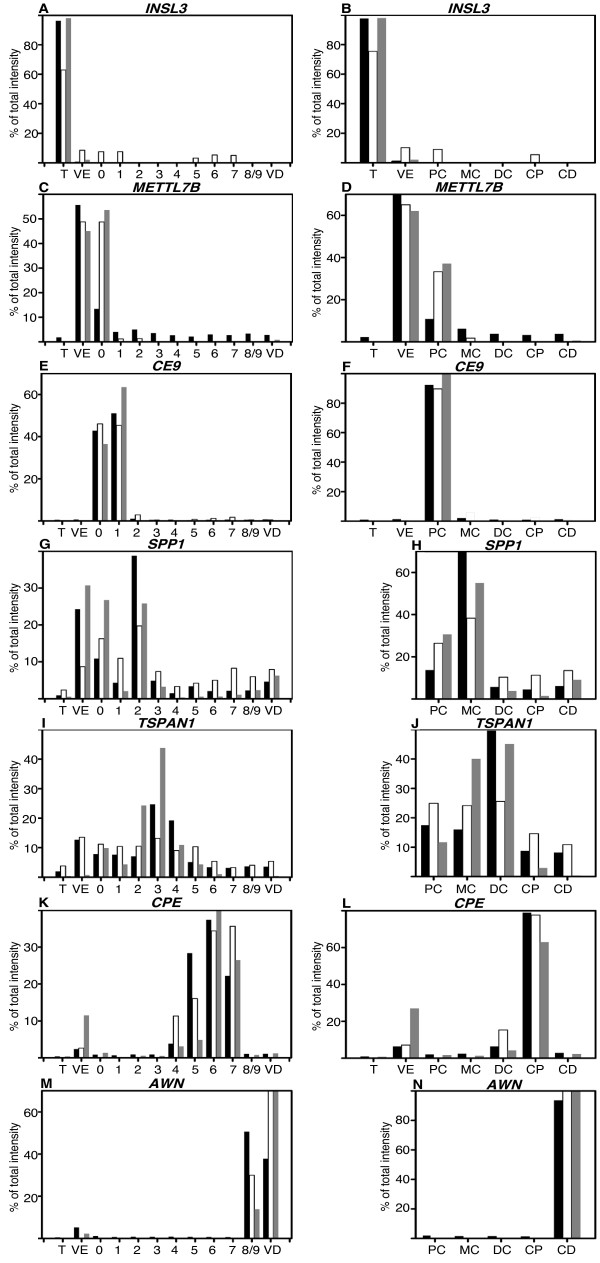
**Comparison of relative expression of selected transcripts on microarray, PCR and realtime PCR**. **A-C-E-G-I-K-M: **Relative expression of selected transcripts obtained from microarray, PCR (from Figure 4) and realtime PCR. Each value is expressed as a percent of the total intensity measured in the 12 samples studied (see materials and methods). In black, microarray values (each data is the mean of the data from the 4 boars studied), in white RT-PCR values and in grey Q-PCR values (values were obtained from one of the boar used in the microarray study). T: Testis, VE: *Vas efferens*, 0 to 8/9: nine epididymal zones, VD: *Vas deferens*. **B-D-F-H-J-L-N: **Relative expression measured in the 12 samples studied were averaged to reproduce either the 7 or 5 transcriptomic units for which some transcripts have been defined as markers. Microarray, RT-PCR and Q-PCR values are in black, white and grey respectively (T: Testis, VE: *Vas efferens*, PC: Proximal caput (E0-1), MC: Median caput (E2), DC: Distal caput (E3-4), CP: Corpus (E5-6-7), CD: Cauda (E8/9-*Vas deferens*).

Among the transcripts, we have also chosen for each one of our transcriptomic units, one gene found as a marker by the statistical analysis (*INSL3*, *METTL7B*, *CE9 *(homologous to putative membrane protein *HE9*), *SPP1*, *TSPAN1*, *CPE*, *AWN*). The expression of these genes was measured by the intensity of the amplicon on gel after RT-PCR on the twelve different tissues (as in Figure 4), and the validity of the statistical analysis compared after averaging the results to reproduce either the seven or the five transcriptomic units where these genes have proved to be over-expressed and classified (Figure 5). The expression level of these genes was also quantified by real-time PCR and all the results compared to the expression values from the microarray (Figure 5). All PCRs and microarrays results were in very close agreements. For *METTL7B*, which was found as a *vas efferens *marker, a slight discrepancy exists between the microarray and the PCR data. This difference came from the fact that among the four boars the one chosen for the PCR showed the highest expression in zone zero (not shown). Meanwhile when the level of PCR expressions is mathematically pooled to reproduce the seven tissue clusters, this gene expression was clearly higher in the *vas efferens *compared to the proximal caput.

### Expression pattern of gene families of interest

Among the transcripts found in the clusters, some of them belong to families that have been previously described in different epididymal studies. So, we further studied the expression pattern in boar of some of these genes and their related family genes found on our array.

#### a) Lipocalins

**Figure 6:**
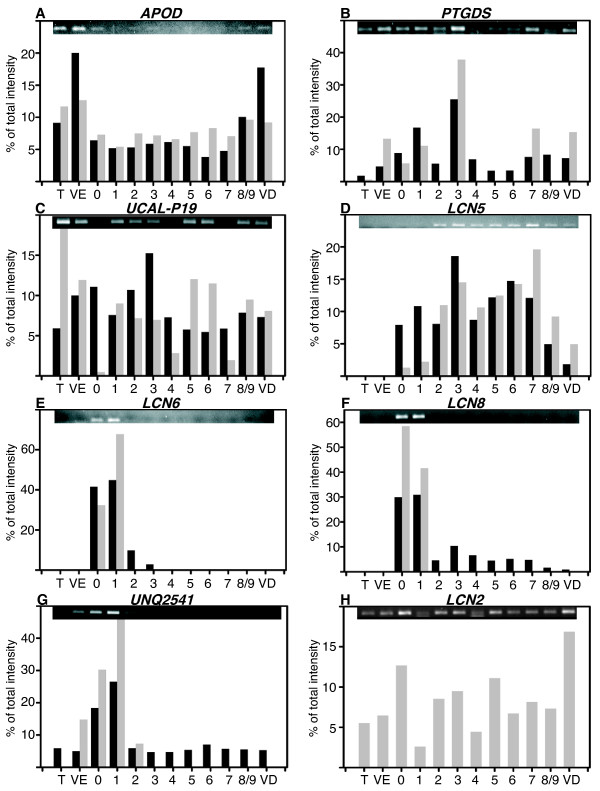
**Lipocalins expression**. **A to H: **Relative expression of the different lipocalins studied: *APOD *(apolipoprotein D), *PTGDS *(prostaglandin D2 synthase), *UCAL-P19 *(uterocalin), *LCN5 *(lipocalin 5), *LCN6 *(lipocalin 6), *LCN8 *(lipocalin 8), *UNQ2541 *(uncharacterized lipocalin UNQ2541/PRO6093 precursor),*LCN2 *(lipocalin 2). On the top of each graphic the image of agarose gel used for amplicon quantifications is shown. In black, microarray values (means of the data from the 4 boars studied) and in grey RT-PCR values. T: Testis; VE: *Vas efferens*; 0 to 8/9: nine epididymal zones; VD: *Vas deferens*. Values represent the percent of total intensity measured in the 12 samples studied (see materials and methods).

Several lipocalin family members have been described in the epididymis and found in the epididymal fluid [36]. We found that clones with related sequences to 7 known lipocalins (*APOD*, *PTGDS*, *LCN5*, *LCN6*, *LCN8*, *UCAL-P19*) and one uncharacterized lipocalin (*UNQ2541*) were spotted on the microarrays and showed a differential expression. We designed primers (Additional File 2) and we compared the results of the microarrays hybridization and RT-PCR expression levels (Figure 6). We obtained a very good matching for *APOD*, *PTGDS*, *LCN5*, *LCN6*, *LCN8*, while the differential presence of *UNQ2541 *was observed in caput with a maximum in zone 1. For *UCAL-P19 *(named uterocalin in horse [37]) a low signal was found all along the epididymis by PCR, suggesting a low level of expression for this gene and the differential expression could not be confirmed. We have also analyzed the *LCN2 *gene expression because this gene is also named Uterocalin in database although there is no sequence homology with *UCAL-P19 *(see discussion). *LCN2 *expression was detected in boar epididymis but did not show a differential expression (Figure 6).

#### b) Defensins

**Figure 7:**
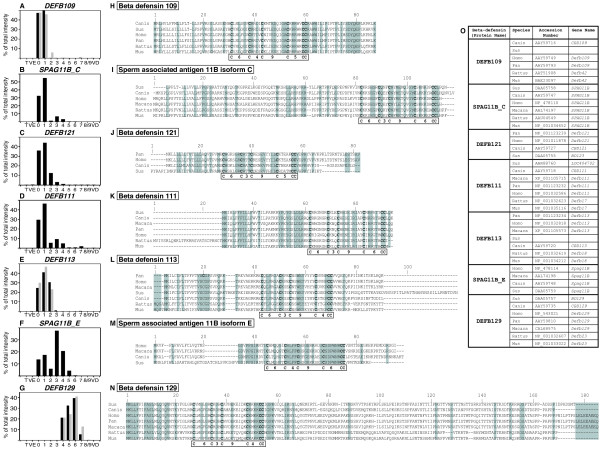
**Relative expression of defensins**. **A to G: **Relative expression of *DEFB109 *(defensin beta 109), *SPAG11B_C *(sperm associated antigen 11B isoform C), *DEFB121 *(defensin beta 121), *DEFB111 *(defensin beta 111), *DEFB113 *(defensin beta 113), *SPAG11B_E *(sperm associated antigen 11B isoform E), *DEFB129 *(defensin beta 129) is represented as the percent of total intensity obtained on microarray (black) and for some compare with the percent of total amplicon intensity measured on the 12 samples studied (Grey). T: Testis, VE: *Vas efferens*, 0 to 8/9: nine epididymal zones, VD: *Vas deferens*. **H to N: **Comparison of the pig deduced amino acid sequences of each beta-defensin from different mammalian species. Amino acid residues that are identical between at least two sequences in alignments are placed in shaded boxes. Cysteins of the beta-defensin domain are in bold and for each protein, numbers of amino acids between the characteristic cysteins are reported in boxes. Hyphens represent gaps introduced for optimal alignment. **O: **Accession number of sequences used for alignments.

Specific expression of a large number of defensins has been shown by previous epididymal transcriptomic studies [20,21,38]. The data mining of the clone sequences spotted on our pig microarray showed that at least 7 defensin genes were present and differentially expressed (*DEFB109*, *DEFB111*, *DEFB113*, *DEFB121*, *DEFB129*, *SPAG11B_C, SPAG11B_E*). The identity of each of these pig defensins was ascertained by sequence comparisons and cysteine positions and spacing (Figure 7H to 7N, and Figure 7O). Five of these defensins were already correctly described in pig (*DEFB111*, *DEFB121*, *DEFB129*, *SPAG11B_C, SPAG11B_E*) and two were miss-annotated (*DEFB111*, *DEFB121*). We also identified two new boar defensins homologous to *DEFB109 *and *DEFB113*.

By RT-PCR, the 7 defensins were found exclusively expressed in the caput and corpus epididymis (Figure 7). *DEFB109 *is mainly restricted to the anterior caput (Figure 7A) while *SPAG11B_C, DEFB121, DEFB111 *genes had a high relative expression in that region with a smaller expression in the posterior caput (Figure 7B–D). *DEFB113 *had a high relative expression in the caput through zones 0 to 2 (Figure 7E). *SPAG11B_E *was expressed in all caput zones with a maximum in the posterior caput epididymis (Figure 7F) while *DEFB129 *(Figure 7G) was found mainly in corpus (zones 5–6) with expression in adjacent zones (4 and 7).

#### c) Whey acidic containing domain proteins (WAPs)

**Figure 8:**
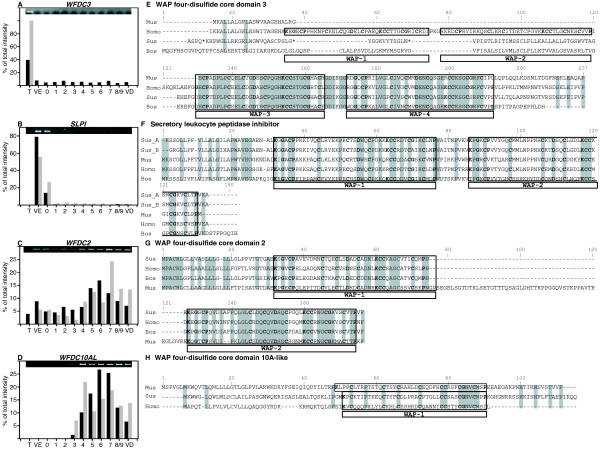
**Protease inhibitors expression**. **A to D: **Relative expression of *WFDC3 *(WAP four-disulfide core domain 3), *SLPI *(secretory leucocyte peptidase inhibitor), *WFDC2 *(WAP four-disulfide core domain 2), *UNQWAP *(uncharacterised WAP four-disulfide core domain protein) is represented as the percent of total intensity measured in the 12 samples studied by microarray in black and by RT-PCR values (grey). T: Testis, VE: *Vas efferens*, 0 to 8/9: nine epididymal zones, VD: *Vas deferens*). On the top the image of the agarose gel used for quantification. **E to H: **Comparison of the amino acid sequences of deduced Whey Acidic Protein family from different mammalian species. Identical Amino acid residues between at least two sequences in alignments are placed in shaded boxes. Characteristic domains are boxed and residues of the WAP domain are in bold. Hyphens represent gaps introduced for optimal alignment. Accession number of sequences used for alignments are: E: Mus (NP_082237), Homo (CAC36106), Sus (unpublished), Bos (XP_001789331); F: Sus_A (translated from scac0031.l.24), Sus_B (P22298), Mus (NP_035544), Homo (NP_003055), Bos (XP_590087); G: Sus (NP_999112), Homo(NP_006094), Bos (NP_001069958), Mus (NP_080599) and H: Mus (NP_001012741), Sus (translated from BX915529), Homo (NP_542791).

Like lipocalins and defensins, WAP protease inhibitors have been found in epididymis [39,40]. We identified *WFDC2*, *WFDC3*, *SLPI *and a new WAP domain sequence that we named *WFDC10A-like *since it shows similarities to mouse and human putative protease inhibitor *WFDC10A *(Figure 8E–H). *WFDC3 *was mainly expressed in the testis (Figure 8A) while SLPI was expressed in *vas efferens *and zone 0 (Figure 8B). *WFDC2 *had a low expression in the testis and all along the epididymis including the *vas deferens *but with a highest level in the corpus and cauda (Figure 8C). The *WFDC10A-like *gene showed a more restricted expression from the posterior caput to the *vas deferens *with a maximum in the distal corpus and cauda (zones 6 and 7; Figure 8D).

### Expression pattern of some new epididymal genes or genes not yet described in boar

#### a) New variants of known genes

**Figure 9:**
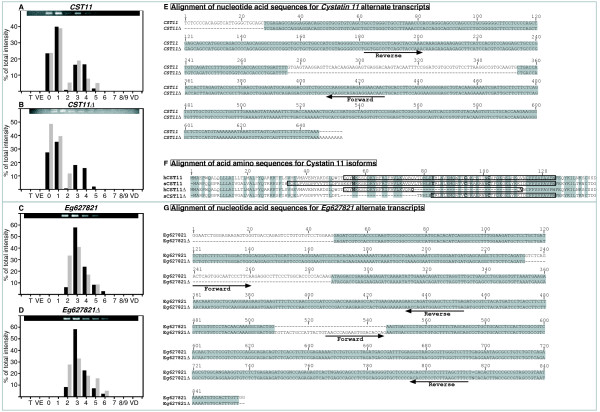
**Gene variant analysis**. **A to D: **Relative expression of *CST11 *(Cystatin 11 alternate transcript 1), *CST11_*Δ (Cystatin 11 alternate transcript 2), *Eg627821 *(predicted gene, *EG627821 *alternate transcript 1),*Eg627821_*Δ (predicted gene, *EG627821 *alternate transcript 2) is represented as the % of total amplicon intensity (grey) and corrected microarray values (black) measured in the 12 samples studied. T: Testis, VE: *Vas efferens*, 0 to 8/9: nine epididymal zones, VD: *Vas deferens*). On the top: image of agarose gels used for quantification. **E: **Comparison of nucleotidic sequences of Sus Scrofa *CST11 *alternate transcripts showing the primer positions used for amplification. Hyphens represent gaps introduced for optimal alignment. Numbers are residue numbers. Accession numbers for *CST11 *and *CST11 *Δ are respectively scac0028.a.13 and scac0036.l.15. **F: **Amino-acid sequences comparison between Sus Scrofa and Homo Sapiens *CST11 *isoforms showing that they have a different deletions. Amino acid residues that are identical between human and pig are shaded. Residues shown to belong to the cystatin domain by Pfam analyses are boxed and those characteristics of this domain are in bold. Accession numbers for hCST11, hCST11Δ are respectively NP_570612 and NP_543020. Amino acid sequences for sCST11 and sCST11_Δ are deduced from scac0028.a.13 and scac0036.l.15 respectively. Hyphens represent gaps introduced for optimal alignment. **G: **Comparison of nucleotide acid sequences of Sus Scrofa *Eg627821 *alternate transcripts showing that both sequence have a different deletion. Arrows show the hybridization site of primers used for amplification. Accession numbers for *Eg627821 *and *Eg627821*_Δ are respectively BX924253 and BX922624. Hyphens represent gaps introduced for optimal alignment.

Several cystatins have been shown mainly expressed in epididymis and *Cystatin 11 (Cst11) *is an "epididymal specific" gene expressed in the caput and corpus region in mouse [41,42]. A variant with a deletion (*Cst11Δ2*) has been suggested from "in silico" genomic sequence analysis but not shown *in vivo *[43]. We found two clones over-expressed in our microarray experiments with matching sequence with *CST11 *and one of these clones had a sequence deletion (Figure 9E and 9F). We designed primers encompassing the deletion and the RT-PCR confirmed the presence of two amplicons at the expected sizes (247 bp and 169 bp respectively). The sequencing of these amplicons confirmed the existence of the two mRNAs. We observed that although *sCst11*Δ had a lower intensity than the full *sCST11*, both mRNAs were present in the same zones with a same biphasic pattern of expression (Figure 9A and 9B).

*Eg627821 *(also named EST AV381130) was described in mouse epididymis as a specifically caput/corpus over-expressed gene [44]. DNA sequence analysis suggested two possible peptide sequences by alternative RNA splicing but only one mRNA was analyzed in this previous study. We found two clones over-expressed showing both a different deletion when compared to the mouse sequence. We have ascertained the existence of both messengers by RT-PCR with specific primers and amplicon sequencing (Figure 9G). We found that both mRNA were equally expressed in posterior caput with a similar expression pattern (Figure 9C and 9D).

#### b) New unreported genes in boar

Among our over-expressed genes we choose two genes that were not previously observed in epididymis and four not yet described in boar.

**Figure 10:**
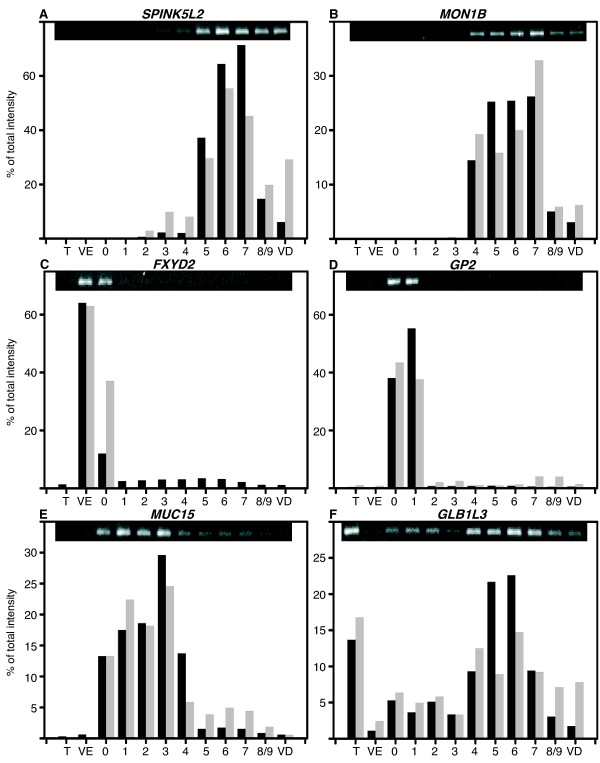
**New genes in boar testis and epididymis**. **A to F: **Relative expression of *SPINK5L2 *(Kazal type serine protease inhibitor 5-like 2), *MON1B *(MON1 homolog B), *FXYD2 *(FXYD domain containing ion transport regulator 2),*GP2 *(glycoprotein 2), *MUC15 *(mucin 15),*GLB1L3 *(galactosidase, beta 1-like 3) is represented as the % of total RT-PCR amplicon intensity (grey) measured in the 12 samples studied (T: Testis, VE: *Vas efferens*, 0 to 8/9: nine epididymal zones, VD: *Vas deferens*) and compare with the normalized microarray intensity values (black). On the top of the graphic the image of agarose gel used for quantification.

*SPINK5L2*, a Kazal type serine protease inhibitor, is a marker of the corpus with a maximum intensity in zones 6 and 7 (Figure 10A). *MON1b*, a new member of the SAND protein family, was also expressed in the cauda (Figure 10B). *FXYD2*, the gamma subunit of the Na, K-ATPase, was found as a marker of the VE (Figure 10C). *GP2*, Glycoprotein 2 (Pancreatic zymogen granule membrane protein GP-2) gene not yet described in epididymis, is highly expressed in the anterior caput in boar (Figure 10D). *MUC15 *is a novel mucin-like protein that is over-expressed in the boar caput epididymis (Figure 10E). *GLB1L3 *(Galactosidase, beta 1-like-3), is a new member of the galactosidase beta family. We found a high expression of this enzyme in the boar testis but also a differential expression in the epididymis with a maximum in corpus (Figure 10F). For all of these genes we find a very good agreement between microarray over-expressions and RT-PCR results.

### Gene Ontology

**Figure 11:**
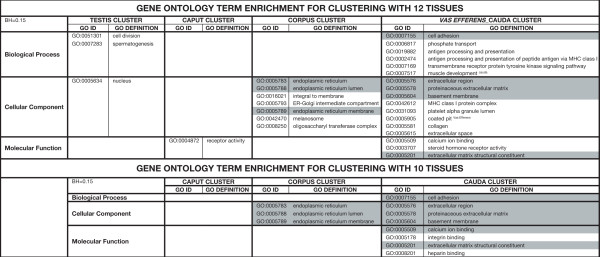
**Gene ontology terms enrichment**. For each clustering analysis, GO terms enriched in one of the 4 or 3 clusters of transcripts are given. Terms that are common of the 2 clustering analyses are shaded. For muscle development and coated pit terms, the unit where the most genes encode for these terms is noticed in exponent.

We have associated the proper GO terms from the Gene Ontology Consortium annotation categories for each gene (biological processes, cellular components, and molecular functions) and generated a list of over-represented GO terms using either the five transcriptomic clusters or only the three epididymal clusters. From this analysis, several over-represented categories were identified (Figure 11). As expected, and as a good internal control, "spermatogenesis" was one of the functions associated with the testis, as well "cell division" and "nucleus". (For list of genes related to these functions see: Additional File 3 and Additional File 4)

### Similar gene expression in mammals

Different epididymal transcriptomic studies have been done in mouse, rat and human. Comparison of the data between rat and mouse have been made and showed the difficulties to compare the different zones of these two mammalians epididymis, even using similar microarray technology [20,45]. In human, the main study was done on three epididymal samples using the head, the corpus and the cauda epididymis but no anatomical description of this division was shown [22].

However, we tried to compare our data with those of these three studies. We have first obtained the list of expressed genes in the different studies. One obstacle to this comparison was the low level of gene annotation in boar or homogeneity of genes annotation between species. To encompass this problem, we have transformed all the genes in their *mus musculus *homologous UniGene IDs. We searched transcripts that have their higher level of expression for the 4 species studied, in the same epididymal unit (caput, corpus, cauda) or in segments corresponding to these units. For mouse, the 3 units were defined based on anatomical and clustering information given by Johnston et al., [21]. The caput was formed from segments 1 to 6, corpus by segment 7 and the cauda segments 8 to 10. For rat, the 3 units were defined from anatomical information given by Jelinsky et al., [20]. The caput was composed by segments 1 to 6, the corpus by segments 7 to 11 and the cauda by segments 12 to 21. For human we used caput, corpus, cauda defined in the Thimon et al., [22] study. These data were compared to the boar over-expressed genes list.

We then found that 768 mouse UniGene IDs were common between the 4 studies. Among these 768 genes IDs, 236 unigene IDs were found over-expressed both in one region of boar and one of the regions of the three other different species (57 in the caput, 131 in corpus and 48 in cauda). Meanwhile when we looked for the common over-expressed genes in the same unit in the four different species we ended with only 8 IDs in the caput, 4 in corpus and 35 in cauda (For the complete list of common gene IDs see Additional File 5).

### Gene deletion effect

Using the mouse homologous IDs found for our markers, we search the MGI database for reproductive phenotypes (Additional File 6). 46 of them were associated with a reproductive phenotype, and 16 with male infertility. A majority of these gene knockouts results in an impaired sperm production associated with a poor sperm quality, suggesting a testicular action. Meanwhile these genes were not all markers of the testis in our study, may be indicating differences between mouse and pig. On another hand, for some genes that were expressed both in testis and in epididymis, the first effect of their knockout might be visible on the testicular function and then affect the sperm formation, and in this case an effect on the epididymal maturation becomes secondary or not visible.

## Discussion

Here we used a 9 K Nylon microarray made during the French AGENAE program which is spotted with 8931 PCR amplified clones. Although this represents only a subset of all genes expressed from the complete genome, one of its advantages is that cDNA clones libraries from the testis and the epididymis were included at the initial step of normalization. Arrays were hybridized to compare gene expression between the testis, *vas efferens*, nine epididymal zones (0 to 8/9) and the *vas deferens*. The pig epididymis is a large organ of more than 20 cm long containing about 50 meters of a convoluted tubule and its internal organization is much complex than in rodent. It is then more difficult to individualize part of the boar epididymis between connective tissue *septae *as it has been done in mouse and rat [20,21]. Doing this will have been impossible in a time-frame necessary to obtain samples from one animal without mRNA degradation and this would have conducted to analyze tens of different epididymal segments which is time and money consuming. Moreover previous study on pig secretome [5,26] and data obtained in rodent suggested that much less transcriptomic units might exist than anatomical separations imply. With pig epididymis it is possible to obtain large samples, including from the *vas efferens *that is more difficult in rodents. Meanwhile anatomical differences between two individual boars may exist, and although we have drawn a special attention to be very reproducible when collecting the samples from different animals, we observed some differences in the gene expressions from some epididymal zones between animals resulting in zones shuffling during membrane clustering (see further). Because acquisition of motility and fertility by the spermatozoa is progressive along the epididymis and secretion of proteins is regionalized, we postulated that genes expressed in a tight regionalized manner might play a major role in this process. For this reason we have focused our attention on differentially expressed genes. In doing this we expected to gain information on how this regionalization is maintained (common transcription factors controlling the genes expressed in one transcriptomic unit), get clues on specific mechanisms (clusters of genes involved in the same physiological function) and deduced factors that may be important in the sperm fertility acquisition and epididymal regionalization. Then we have not analyzed major known epididymal genes such as clusterin or PTGDS since we already known that these genes are widely expressed or present at several levels of the genital tract. These genes that are over-expressed in more than one epididymal transcriptomic unit may have important roles for the cellular functions but may be not directly related to the sperm maturation. Meanwhile the microarray approach we used offer the possibility to analyze also these genes, since they could be retrieved in the list of expressed genes that is deposed on the GEO database (GSE15614).

Because our type of array is not widely used and the biological question more complicated than a simple comparison between two conditions on the same tissue, few statistical tools were available. We had then adapted classical microarray statistical approaches in order to compare the different conditions with a low number of replicates. We have also decided to cross the results from different statistical analysis to ascertain and strengthen our results, especially to define epididymal markers. Our analysis found 2115 transcripts with a differential expression between the 12 conditions; this represents about 24% of the transcripts spotted and 27% of our 7676 qualified spots. In rat about 13% (4000 genes) from the 30000 transcripts of the microarray were found highly regulated along the epididymis and comparable results were obtained in mouse and human.

Tissues clustering indicated a good biological reproducibility since arrays from the same tissues from different animals clustered together with few exceptions where one zone could shift from one cluster to the previous or the following one. We did not observe an effect of the breed induced by the 3/4LW-1/4Landrace boars. The clustering procedure builds 7 informative tissue array clusters from our data. The testis was always well separated from the other tissues and the epididymal zones were clustered in anterior-, median-, posterior-caput, corpus and cauda (including the *vas deferens*). The *vas efferens *formed one independent cluster.

In contrast, transcripts clustering defined only 4 different co-expression clusters: testis was separated from the rest of the tissues then *vas efferens *and cauda/*vas deferens *were grouped together while two epididymal clusters, caput and corpus, were formed. This clustering difference (7 tissue clusters against 4 co-expressed transcript clusters) could be explained by the number of objects included in each analyze: for classifying the 48 microarrays the procedure used 2115 transcripts, while transcripts were classified using only 3 or 4 values for each of the 12 different tissues which reduced the statistical power of the analysis.

In that analysis *vas efferens*/cauda/*vas deferens *may have been clustered together because they have some common genes involved in common functions (see GO terms) such as ions binding and transport, or response to the same regulatory factors (for ex: retinoic acid receptor *RARRES2 *or steroid hormone receptor) or more strikingly gene such as *AWN *that codes for a specific boar sperm associated protein described mainly in the seminal vesicle [46], although it was also immunolocalized in rete testis and on epididymal sperm [47]. Meanwhile the *vas efferens *showed also a unique and different set of genes expression in agreement with its mixed embryonic origin (embryonic glomerulus and upper Wolffian duct) compared to the epididymis (upper Wolffian duct) and the *vas deferens *(middle Wolfian Duct) [35].

This discrepancy in the number of clusters between tissues and transcripts has conducted us to analyze the data with particular statistical approaches to assign specific differential transcripts for each of the 7 groups of microarrays. The use of two different approaches has also allowed us to strengthen our results and choose the transcripts with more confidence. Moreover the search for differential genes in each epididymal transcriptomic unit brought us to re-do the analysis excluding testis and VE units. In doing that, we increased the number of transcripts in some of the epididymal clusters but this was not general since some clusters have also lost some genes that became non statistically significant over the epididymis as they were when the testis or the *vas efferens *were included. This approach also explains why some of the gene known to be highly expressed by the boar epididymis were not found in the list of markers; these genes were either expressed in two different clusters (ie testis and epididymis) or their high expression spanned over different adjacent clusters and then they were not retained as differential between the epididymal transcriptomic units. This was the case for example for RNase 10 (formerly described as train A), previously found in the pig anterior caput [16], that was found over-expressed in our array analysis exclusively from zones 0 to 2 then in both anterior and median caput clusters.

We have ended with lists of significant over-expressed transcripts and markers for each of the tissue clusters that were further used to validate our microarrays data. For almost all transcripts chosen, we find a very good agreement between the level of expression obtained either by microarrays, RT-PCR and real time PCR. Among these genes used for this validation we have selected a certain number because information on expression in male reproductive tract were available and we have also analyzed new genes and genes not yet reported in epididymis.

The first one was clusterin (*CLU*). The mRNA is present both in the testis and the epididymis as well as different isoforms of the protein. This protein as many different functions (chaperone, lipid transport etc) but its exact role in these organs is not yet known. This dual expression was retrieved in boar by microarray and PCR, and *CLU *was classified in our analysis in the testis due to its higher level of expression in this tissue. This gene is expressed over more than one transcriptomic unit of the epididymis (distal caput and corpus) where the protein secretion and presence in the fluid has already been described [16,26]. *INSL3 *and *INHA *were genes already known to be testicular and were found over-expressed in the boar testis. *INSL3*, expressed mainly in Leydig cells, mediates the first phase of testicular descent and mutation of this gene leads to cryptorchidism [48]. *INHA *encodes for the inhibin alpha subunit, mRNA and protein chain have been localized to Sertoli cells, which is the major body source of this protein.

For the anterior caput, the boar homologue to *CE9 *(canine epididymal 9 gene) was analyzed. This gene has been found in the upper part epididymis of dog and mouse epididymis (named *ME9*) but not in human epididymis [49,50]. It has been suggested that its expression is regulated by luminal factors from the testis [51]. This gene encodes for an integral membrane protein and no clear function as been yet assigned.

*GPX5 *gene has also been extensively study in the epididymis where it codes for an androgen-dependent glutathione peroxidase specific in the distal caput epididymis of mouse and rat [49,52]. A role for sperm and epididymis protection against oxidative stress has been suggested for this enzyme. In pig this mRNA was shown caput epididymis specific where the protein has been found secreted in the fluid [26,53]. In agreement with these results we found *GPX5 *over-expressed in zones 0 to 3. Secreted Phosphoprotein One (*SPP1*, Osteopontin) is widely distributed and was found over-expressed in *vas efferens *and caput and at a lower level in cauda epididymis. This protein is involved in different cellular processes such as calcium transport, bone resorption and extracellular matrix (ECM) remodeling. In rat, it has been expressed and localized in testis, *vas deferens*, and caput epididymis where it was found associated with cellular vesicles and may be involved in fluid calcium depletion [54]. In bull seminal plasma, this protein has been related to sperm fertility [55]. In boar polymorphisms in the *OPN *gene region have been significantly associated with male fertility traits, mainly motility and acrosome reaction [56].

*MAN2B2 *is a lysosomal type D-mannosidase secreted in porcine epididymal fluid (135 kDa alpha-D-Mannosidase) [57,58] and was found expressed and secreted only in a narrow region between the caput and corpus epididymis while it is found mainly in the murine testis at the spermatogonia level [59]. Recently a polymorphism in this gene was associated with ovulation rate in a Meishan-cross gilts population [60]. Our data confirmed that *MAN2B2 *was mainly epididymal in boar with a caput-corpus specific over-expression. Recently we have described the *CES7 *carboxylesterase (also named cauxin for carboxylesterase-like urinary excreted protein) in the epididymal fluid of various mammals, and its mRNA was found in ram corpus epididymis. Here we showed that like in ram, *CES7 *expression is restricted to the corpus epididymis where the protein was immunolocalized in the boar fluid [17]. Carboxypeptidase E (*CPE*) is a pro-hormone processing enzyme (such as pro-insulin, pro-gonadotropin-releasing hormone and pro-opiomelanocortin) in neuro/endocrine cells and a peripheral membrane protein of secretory granules where it plays also a receptor role that directs the pro-hormone to the regulated secretory pathway [61,62]. The expression of *CPE *mRNA in both neural tissues and some non-neural tissues has been shown during embryonic development [63] but not reported in the reproductive tract. Interestingly *CPE *gene is over-expressed in the corpus epididymis where the main enriched GO terms are related to reticulum endoplasmic functions. Whether this region secreted specific factors or hormones that need to be processed by carboxypeptidase E remains to be elucidated.

We have also focused our attention on several gene families that have been previously described in different epididymal studies.

Lipocalins are found in vertebrates, insects and plants. They are involved in the transport or storage of hydrophobic and/or chemically sensitive organic compounds, especially vitamins, lipids, steroids and other secondary metabolites [64]. Several lipocalins have been described in epididymis such as the retinol-binding protein *LCN5 *(epididymal retinoic acid-binding protein, E-RABP)), *LCN6*, *LCN8*, *LCN9*, *LCN10 *or the *PTGDS *(prostaglandin D2 synthase) and numerous studies have shown their regional intensities of expression both at mRNA and proteins levels [65]. Their exact individual role in the epididymal function is still unclear. Here we have studied some of these lipocalins (*LCN5*, *LCN6*, *LCN8*, *PTDGS*) and shown their regional expressions in boar and we have described new lipocalin members that were not yet observed in mammalian epididymis (*UCAL-P19 *and *APOD *(apolipoprotein D)) and a new lipocalin member matching with the human *UNQ2541 *sequence.* UCAL-19 *is a 19 kDa lipocalin that has been described in the horse endometrium before embryo attachment where it is secreted abundantly suggesting a role between maternal and foetal tissues dialogue [37]. This gene was improperly name uterocalin since it has a different sequence from mouse uterocalin (*LCN2*, neutrophil gelatinase-associated lipocalin NGAL) which is a 25-kDa lipocalin originally purified from human neutrophils and highly expressed in rodent caput epididymis [65,66]. In boar epididymis *UCAL-P19 *showed a differential expression by microarray that could not be clearly confirmed by RT-PCR, certainly due to its low level of expression. PCR indicated also that, contrary to rodent, LCN2 had a low and not differential expression in boar. Apolipoprotein D (*APOD*), a 29-kDa glycoprotein associated with high-density lipoproteins, is predicted to be a member of the lipocalin family based on its primary structure [67]. The protein is multifunctional and has many different ligands. It binds and transports small hydrophobic compounds including sterols-derived molecules. Its mRNA has been localized in numerous tissues including the testis. We retrieved the testicular expression of this gene and showed that it is also expressed more in the *vas efferens *and *vas deferens *than other epididymal regions. Human *UNQ2541 *sequence, for which no role or localization information is available, was located on chromosome 9 close to a lipocalins cluster containing *LCN6*, *LCN8 *and *LCN10*. Its expression was very similar to *LCN6 *and *LCN8 *expression in boar caput epididymis suggesting they could be controlled by the same factors [36].

Defensins, a family of secreted antimicrobial cationic peptides with molecular weights of 4–5 kDa, have a distinctive sequence pattern of six to eight cysteine residues. They are categorized into alpha-, beta-, and theta-defensins according to the arrangement of their three-four disulfide bonds [68,69]. Defensins peptides are the major antimicrobial proteins of innate immunity found in mammals and it seems that in mammals defensins are mainly from the alpha and beta classes. In human over 40 beta-defensins ORF have been found (but only 4 characterized at the peptide levels) and in rat and in mouse at least 42 and 52 genes and pseudogenes respectively were discovered by genome scans [70]. Moreover, it is also demonstrated that some defensin gene (such as *SPAG11*) are able to use different exons to produce multiple isoforms of beta-defensin-like sequences [71]. Specific expression of numerous epididymal beta-defensins has been shown and SPAG11 and recently DEFB126 have been found associated with sperm and the latter is involved in attaching sperm to the oviduct epithelia and may be involved in sperm motility and capacitation [72,73]. In their study Jelinsky et al. [20] analyzed 29 defensins by QPCR in mouse and rat and showed that they had very similar epididymal regionalization in these two species. Recently, Sang et al. [74] in a blast search on pig EST described 12 beta-defensins and eight were found by RT-PCR in the male reproductive tract including the epididymis (caput, corpus, cauda) and the testis. *pBD4*, *pBD108 *were found mainly in proximal epididymis while *pBD3 *was mainly in cauda. *pEP2C *was found in testis and epididymis while *pBD104 *and *pBD2 *only in testis. Other defensins were also expressed (*pBD114*, *pBD123*, *pBD129 *(*DEFB129*), *pBD125*) in testis and/or epididymis but without differential expression in this last tissue. We have ascertained the identities by sequencing and cysteine motifs alignments of the seven beta-defensin clones found spotted on our microarray. Five of them showed an anterior caput over-expression while the *SPAG11B_E *isoform had a posterior caput expression. Only *DEFB129 *was found in corpus. The discrepancy with the previous study could be explained by the fact that these authors used a 45 cycles RT-PCR that is certainly not as discriminative as our microarray analysis. Moreover for this defensin and the two other (*DEFB109 *and *DEFB113*), that were also analyzed by RT-PCR, we found a very good agreement with the microarray data. Interestingly the expression profiles of the defensin genes in boar were closely similar to those observed in mouse and rat, even for the *SPAG11B *isoforms.

Proteases and proteases inhibitors have an important role in proteins processing both in tissues, fluids and at the sperm plasma membrane levels during the maturation process. We have focused on several members of different type of protease inhibitors. WAP protease inhibitors contain the WAP four-disulfide core domain structure, a motif first described in whey acidic protein present in the milk of rats and mice. WAP motifs consist of about 50 amino acids with eight highly conserved cysteine residues forming four disulphide bridges. Then WAP protease inhibitors possess like defensins specific cystein motifs and exhibit antimicrobial and antifungal activities (may be due to this wap domain) indicating also a role in the innate immunity [75,76]. Several inhibitors of this family have been found in epididymis [39,77] such as *WFDC2 *(WAP four-disulphide core domain protein 2, HE4 [78,79]) and *SPINLW1 *(serine protease inhibitor-like with Kunitz and WAP domains 1, EPPIN [80]) and may play a role in sperm maturation and function. *WFDC2 *(HE4) is also over-expressed in different normal and malignant tissues [79]. We found several wap domains proteins in the clones spotted and we ascertained their identity by sequence comparison. *WFDC2 *was mainly found in caput epididymis in mouse and rat and it is a distal cauda epididymal gene in human. In boar, it is present all along the epididymis with an over-expression in cauda. *WFDC3 *is a testicular over-expressed gene in boar while it is found in testis (round spermatids) and highly expressed in caput/corpus in rat. SLPI is expressed in wide range of tissue and it has been immunolocalized in human epididymis and found in seminal plasma. It has been shown highly expressed all along the rat epididymis and slightly in mouse [20,21]. In boar, *SLPI *appears as a specific *vas efferens *gene. We analyzed also an uncharacterized WAP domain-containing protein with an unknown function mainly expressed in the boar cauda epididymis and that was named *WFDC10A-like *from the closest human sequence found (*WFDC10A*). The exact role of all these wap domains proteins as inhibitors in epididymis remained to be elucidated as well as their potential role as bacterial defenses. Interestingly, *WFDC2*, *WFDC3*, *WFDC10A *and *SLPI *are found on the same gene cluster in human chromosome 20. The mouse *WFDC10 *homologuous gene has also been found highly expressed in mouse cauda epididymis [39].

Because a large number of gene are translated in several spliced mRNAs coding for different proteins and this different spliced mRNAs may be differentially expressed along the epididymis (i.e *SPAG11*), we analyzed two genes for which spliced mRNA forms were found in our data.

Cystatins are reversible inhibitors of cysteine proteases. They are found in all living organisms and are involved in various biological processes and also take part in disease pathology [42,81]. Several cystatins have been shown mainly expressed in epididymis such as *Cst8 *(CRES) in proximal caput where it is secreted in the fluid [82]. Cystatin 11 (*CST11*) known as CRES2 in mouse is a member of the CST type 2 family. Its mRNA was identified in Macaca epididymis where it is expressed almost exclusively in the epididymis. The protein is most abundant in the initial segment, but is detected throughout the epididymis and on ejaculated sperm [43]. These authors indicated that a spliced deletion form of this mRNA could exist (*Cst11Δ2*) and both recombinant proteins were produced. Interestingly both form of this protein exhibited antibacterial activity. We found the full length and the deleted mRNAs in our spotted clones, and observed by PCR that both mRNA exist and were expressed in the epididymis. Both had a very similar pattern of expression with two maximum (proximal caput and caput/corpus junction) although the deleted transcript showed lower expression intensity. This caput over-expression of *CST11 *is also reported in mouse and in rat.

We also found in our microarray two clones that were similar to the mouse *EG627821 *mRNA (previously EST *AV381130*) that has been found in mouse epididymis [44]. This gene has an unknown function. In pig, this sequence matched two clones, both showing a different deletion when compared to the mouse sequence. Both mRNAs were ascertained by PCR and sequencing, they showed similar level and pattern of expression in posterior caput/corpus. The mouse mRNA was shown expressed in caput epididymis only after puberty by real time PCR and confirmed by transcriptomic study [44].

At least we have also analyzed newly discovered genes and genes not yet reported in boar. The first one was *SPINK5L2*, which is a putative Kazal type serine protease inhibitor 5-like 2. SPINKs are a family of inhibitors with a characteristic signature consisting of six consensus cysteines forming three specific disulfide bonds [39]. Several members of this inhibitor family have been shown to have a differential expression in the mouse, the rat and the human epididymis. Their protease targets in the epididymis remained elusive. Recently, as part of a cluster of SPINK, five new SPINK members were identified including *SPINK5L2 *[83]. None of these members had functional annotation. We found that the mRNA of the pig *SPINKL5 *homologue is highly expressed in the corpus epididymis. We found also a gene that is similar to MON1B, a SAND family protein. These proteins have recently been shown to function at the tethering/docking stage of the vacuole fusion as a critical component of the SNARE complex [84]. The MON1B gene is found over-expressed in the corpus epididymis were it is known that a high level of secretion takes place. *FXYD2*, also known as the gamma subunit of the Na, K-ATPase, regulates the properties of that enzyme. In mouse epididymal transcriptome this gene was not reported and in rat it was found mainly over-expressed in the cauda. In boar it is mainly in the VE where it may play a role in fluid/ion balance.

*GP2*, Glycoprotein 2 (Pancreatic zymogen granule membrane protein GP-2) gene encodes for a GPI-linked membrane protein that plays an important role in assembly of vesicular compartments targeted to apical plasma membranes via regulated exocytosis. This gene was not found differentially expressed in mouse, rat and human epididymal transcriptome. *MUC15 *is a novel mucin-like protein that showed structural characteristic of membrane-bound mucins including an extracellular region with several potential glycosylation sites, a putative transmembrane domain, and a short cytoplasmic C-terminal. mRNA presence has been reported in human testis [85] and transcriptomic study had shown it in human corpus epididymis [22], but it was not reported in mouse and rat epididymis. The galactosidase *GLB1L3 *mRNA was not previously reported in epididymis but it is mainly found expressed in testis and prostate using the ESTProfile viewer at NCBI. The protein has recently been reported in mice acrosome by proteomic study [86]. In boar it is present in testis and shows a regional expression in corpus. This glycan-modifying enzymes may play either a role in degalactosylation of sperm surface and/or fluid glycoproteins observed during epididymal transit [7].

The use of microarrays allows also analyzing differential functions through the use of gene ontology terminology. In boar it was difficult to use all the information due to the few genes with a valid GO annotation. We had to transform genes in their mouse, human, rat, dog or bull homologous genes and retrieved when possible the GO term associated. This results in the loss of a large number of transcripts. However we were able to found several functions over-represented in the different transcriptional units when we compared them together. We analyzed the functions either with four clusters including testis and *vas efferens *or only with clustered transcripts in the three epididymal units. In testis our GO analysis revealed function related to gametogenesis, this is not surprising but gave a good indication on the validity of our data and analysis. As already described some function were common between the *vas efferens *and *vas deferens *(fluid and ion transports), but the cauda and *vas deferens *had functions specific and related to the extracellular matrix remodeling. This enrichment in these GO terms has also been observed in human, mouse and rat, suggesting that an important plasticity of this region exists, may be related to the storage of sperm in that part of the epididymis. In the corpus the main terms were associated with genes involved in the endoplasmic reticulum function and membrane/vesicles trafficking, suggesting protein synthesis and secretory/reabsorbtion pathways. In boar, immunological and histological studies have described the different pathways of secretion and reabsortion of vesicles at the cellular level [5]. Although different types of vesicles and mechanisms are involved in this process, a well-developed Golgi apparatus system is mainly present in distal corpus were the secretomic analysis had shown that the most intense proteins are secreted and the number of secreted proteins increases from the caput to the caput/corpus region. This Golgi apparatus is still present in the corpus where the number of secreted proteins remains high. The finding that differential genes expressed between caput and corpus with this GO term may imply that a differential type of protein modifications occurs between these two adjacent segments as it has been suggested by the nature of the secreted proteins [5,15].

In the caput the main enriched term was receptor activity. Receptors for different modulators such as activin, progesterone and FGF were present and among the 9 receptors forming this category at least four were retinoic acid responders. This is in agreement with previous studies in rodent that have shown the important role of acid retinoic in the functions of the caput epididymis [87]. Although *vas efferens *was clustered with cauda and *vas deferens*, the GO enriched term "coated pit" was associated with the VE. This term was related with four different transcripts over-expressed in this tissue with homology to DAB2_HUMAN, LRP2_HUMAN, LRP1_HUMAN and CUBN_MOUSE. LRP1 and LPR2 are members of the LDL receptor family that plays diverse roles in various biological processes including lipoprotein metabolism, degradation of proteases, activation of lysosomal enzymes, and cellular entry of bacterial toxins and viruses. The presence of low-density lipoprotein receptor-related protein-2 (LPR2) has been shown in mouse where LPR2 plays a role in endocytosis of testicular clusterin by the *vas efferens *[88]. Other testicular protein could also be removed by this mechanism such as transferrin or apolipoproteins.

We noticed that three of the four genes (*DAB2 *(disable 2 protein), *LRP2 *(megalin) and *CUBN *(cubilin)) are involved in vitamin D uptake, which is considered as a steroidal hormone. The vitamin D metabolite (25(OH)D3) is synthesized in the liver and circulate in complex with its binding protein (DBP). The messenger for this protein is present in boar testis and zone 2 of the epididymis (Guyonnet et al., unpublished). The 25(OH)D3-DBP complex is internalized via receptor-mediated endocytosis, which is absolutely dependent on the membrane receptors megalin (LPR2), cubilin (CUBN) and the adaptor protein disabled-2 (DAB2), the expression of these proteins is in turn modulated by retinoids [89]. Once in the cell, the biologically active metabolite of vitamin D, (1,25-dihydroxyvitamin D3; (1,25(OH)2D3)) is formed by action of the 1-alpha-hydroxylase (CYP27B1, a cytochrome P450 enzyme) and binds to the vitamin D receptor (VDR). We found that *CYP27B1 *is expressed all along the boar epididymis and *VDR *is expressed in the boar testis, *vas efferens *and epididymis (Guyonnet et al., unpublished). VDR has also been shown in mouse, rat and rooster epididymis [90] as well as the intracellular vitamin D binding interacting protein (Vdrip) that mediates anchors of the complex to the estrogen receptor (ER) and other nuclear receptors [91,92]. VDR can form homodimer but also heterodimer with the retinoid X receptor (RXR) present in the boar *vas efferens *(Guyonnet et al., unpublished) and then regulates genomic expression of a large number of genes. Vitamin D plays a pivotal role in maintaining serum calcium homeostasis by modulating calcium/phosphate absorption via its action on calcium binding proteins, such as *SPP1 *(osteopontin) that is over-expressed in boar VE and caput epididymis. Vitamin D is also a regulator of the aromatase in female and male gonad but also in the epididymis [91,93,94]. Interestingly ions and about 90% of the testicular fluid reabsorbtion occur in the *vas efferens *region, and control of these mechanisms by estrogen has been demonstrated since ERalphaKO mice are infertile primarily due to a defect in efferent ducts development and function related to fluid reabsorbtion [95]. Recently, it has also been demonstrated that (1,25(OH)2D3), stimulates the innate immune response in antigen-presenting cells, like macrophages and dendritic cells but also in other cell types [96]. The 1,25-D/VDR complex bound to the retinoid X receptor increases the endogenous defensin and cathelicidin genes production [97,98]. At least, it was shown that male rat with low vitamin D diet had a lower fertility due to lower testicular sperm production but also poor sperm quality [99].

All along this publication we referred to the previous rodents and human epididymal transcriptomic studies in order to compare the gene expression. We have developed bio-informatic tools to compare information on gene over-expression in the four different species and found that a certain number of genes were over-expressed in the four species in one of the three gross anatomical epididymal regions. This low number of common genes in a similar region was not too surprising. In their study Jelinsky et al, [20] whom made a detailed comparison on both the mouse and rat epididymal transcriptomes were strongly limited due to the identification of orthologous sequences present on arrays. Starting with more than 30.000 transcripts, they "only" found 6198 orthologous genes; 3481 genes were determined to be expressed in the epididymides of the 2 species and 492 were differentially expressed. Among these 492 genes, 380 had similar expression patterns in mouse and rat, and 112 were different.

Although we had a low number of common genes between species, we analyzed the GO term associated with the genes of each regions and found that in the caput six genes formed a cluster with functions in cell and to cell signaling and interaction (including some genes controlled by RXR), in the corpus three genes with cell morphology, assembly and development while in the cauda, two main groups of genes formed first a network in cell to cell signaling, morphology and connective tissue development (24 genes) and a second including 11 genes involved in cell death and endocrine system. This demonstrated, if needed, that the epididymis is highly regionalized and this regionalization, regulated by the endocrine system, shows also an important plasticity.

We did not attempt to do the same analysis with the testicular transcripts with different species. Meanwhile, although both approaches were quite different, we compared the list of the most intense transcripts from our study with the one recently published in a differential boar testis transcriptomic study between two breeds [29]. Among the 50 most intense differential genes reported by these authors, we found only 5 transcripts matching the top 50 most intense testicular transcripts of our analysis (*CYP17A1*, ferritin light and heavy chain (*FTL *and *FTHI*), *CYB5*, *DHRS4*).

## Conclusion

In conclusion, the present study and transcriptomic studies in other species clearly demonstrated that the high degree regionalization in epididymis is linked to the regionalization of gene expression. This is also well in agreement with the previous secretomic and fluid proteins analysis. This regionalization appears as the results of a specific and subtle regulatory mechanisms from the endocrine system involving androgens and also estrogens. Different studies suggested also a numbers of other regulatory components such as retinoid derived from vitamin A. Our study indicated that the *vas efferens *and the caput might have a certain number of genes regulated by retinoid receptors. Moreover our analysis suggests also that secosteroids (such as vitamin D) certainly carried by the testicular and epididymal fluid may have a role in the efferent ducts and epididymal function. It is interesting to note that in rat, epididymis presents the second body level of this compound just behind the kidney [100]. Then the relevance of the vitamin D as an intracrine-autocrine-paracrine system in the epididymis needs to be further analyzed.

All the transcriptomic studies have found that family of genes such as defensins or different type of protease inhibitors are over-expressed in different places of the epididymis. These proteins have role in the innate immunity response against infection from bacteria and fungus. The presence of such large amount of these compounds may be a necessity to reduce or prevent the presence in the fluid or within the spermatozoa from cells of the immune system that could after developed autoantibodies deleterious for reproduction. It is likely that different defensins are much more than antimicrobial, and further functional work will uncover different roles for what is increasingly regarded as a multifunctional gene family. Anti-proteases may also be involved in the regulation of the sperm and fluid proteins processing that occurs during maturation and are part of this maturation process.

At least all these transcriptomic studies allow us to see through the high complexity and specialization of epididymis and differences between species. Numerous genes are under the same common regulators and then could be targets for future studies to understand the mechanisms involved in epididymal regionalization and those implicated in sperm maturation.

## Methods

### Animals

Four adult boars (2 Large White and 2 cross-breed Large White-Landrace (3/4; 1/4)) aged from 18 to 36 months were used in this study (average weight was 301 ± 17 Kg). These animals were provided by insemination centers where they were used for genetic improvement. For each animal, different criteria of the semen quality (volume of semen, spermatozoa concentration, spermatozoa mobility) were followed during their reproductive life and considered as good or very good. Their fertility calculated on at least 150 artificial inseminations ranged from 88 to 94% (fertility 91.8 ± 3.2%; litter size 13.5 ± 0.2).

### Sample collection

Experiments on animals were conducted according to the French laws on animal experimentation (Authorization 37-081). The animals were sacrificed at our local slaughterhouse and the testis and epididymis were removed after bleeding. The testicle and epididymal weights were 626 ± 53 g and 135 ± 7 g respectively. The tissues were carefully dissected in less than 30–40 min after the death of the animal in order to minimize the mRNA degradation. The maximum of interstitial tissue and blood vessels were removed from the tubule. Samples from the testis, *vas efferens *(VE), nine define epididymal zones (zero to 8/9) and *vas deferens *(VD) were snap frozen and used further for mRNA extraction (Figure 1). The epididymal zones were identified anatomically according to morphological and physiological criteria [7]. At the morphological scale, changes in the diameter of tubules and cellular composition of epithelium along the different zones have been observed corresponding at the physiological level, in differences in secretion and absorption activities.

### RNA extraction

Frozen tissue (150 to 200 mg) was homogenized in 2 ml of Tri-Reagent (Sigma, St Quentin Fallavier, France) using an Ultra-Turrax. The RNA extraction was performed according to the manufacturer's instructions with small modifications. A second extraction was performed on the aqueous phase to optimize the RNA quality. For all samples, RNA quantity and quality were measured by absorbance ratio at 260/280 nm (NanoDrop, Thermo Fisher Scientific, Villebon-sur-Yvette, France) and the integrity of the mRNAs was determined by both standard agarose gels and mRNA-chip gel system using an Agilent Bioanalyzer (Agilent Technologies, Massy, France). Only samples passing these tests were kept and for sample that failed, extraction was redone on a different piece of sample in order to meet these criteria of quality.

### cDNA microarray

The set up of the AGENAE cDNA library and of the 9 K cDNA nylon microarray used in this study has been published in detail previously [30]. The microarrays were obtained from the resource centre GADIE (INRA-Jouy-en-Josas, France). Briefly, a pig multi-tissue cDNA library from 38 tissues was used to generate the cDNA microarray. The libraries were constructed, normalized and subtracted following the protocol of Soares et al., [101]. 8931 different pig clones were selected either from the generated multi-tissue library (7749), or USDA clones (835) or from different homemade libraries (347). The cDNA clones obtained after PCR amplification were spotted onto Hybond N+ membranes (7.5 × 2.5 cm) as previously described [102]. Controls were also included: 78 controls (64 waters and 14 empty wells), 195 spikes (193 luciferase, 2 Arabidopsis thaliana clones) and 12 empty-vector controls. The array is published on the site Gene Expression Omnibus  under the reference number GPL3729.

### Labeling and hybridization

Forty-eight RNA samples originating from the four animals were used for microarray hybridization according to the following procedure. A first hybridization was performed using a ^33^P-labelled 19-mer oligonucleotide (CACTATAGGGAATTTGGCC), which matches a sequence present at the extremity of each PCR product to estimate the amount of cDNA dropped on each spot. Only membranes with a good pattern of spotting were retained and were attributed at random for the hybridization with the ^33^P-cDNA from the tissues. After stripping (3 hours 68°c, 0.1× SSC, 0.2% SDS), hybridizations were carried out as previously described [103], with minor modifications. Arrays were prehybridized for 4 hours at 65°C in hybridization solution (5× Denhardt's, 5× SSC, 0.5% SDS). Labelled cDNA probes were prepared from 5 μg of RNA by simultaneous reverse transcription and labeling for 2 hours at 42°C in the presence of 30 μCi alpha ^33^P-dCTP, 2.4 μM dCTP, 0.4 mM each dATP, dTTP, dGTP, 40 units RNasin^® ^Ribonuclease Inhibitor (Promega, Charbonnières-les-Bains, France) and 200 units MMLV SuperScript RNase H-reverse transcriptase (Gibco-BRL, Cergy Pontoise, France) in 30 μL final volume. RNAs were degraded by treatment at 68°C for 20 min with 1 μL 10% SDS, 1 μL 0.5 M EDTA and 3 μL of 3 M NaOH, and then equilibrated at room temperature for 10 min. Neutralization was done by adding 10 μL 1 M Tris-HCl, 3 μL 2 N HCl, 2 μL of an 80 polyA oligomers (1 μg. μl^-1^) and 50 μL of hybridization solution then by incubating during 5 min at 99°C and 1 hour at 65°C.

Arrays were then incubated with the corresponding denatured labeled cDNA for 18 h at 65°C in hybridization solution. After 3 washes (1 hours 68°C, 0.1× SSC, 0.2% SDS), they were exposed to phosphor-imaging plates (24 or 48 hours) before scanning using a FUJI BAS 5000 (Fujifilm France, St. Quentin en Yvelines, France). Each array image was inspected using AGScan software [104,105] to eliminate (flag) "bad" spots and artifacts (dust, etc.) from the analysis. Signal intensities were then automatically quantified. One of the advantages to use radiolabeled complex probes is that they allow detecting very low-expressed mRNAs even when using small total RNA amounts [102,103].

### Microarray data processing

Spots for which the vector oligonucleotide data had intensities lower than a cut-off value established as a mean level intensity of water control spots plus 3 times the standard error deviation were excluded from analysis. This cut-off value was calculated for each microarray independently. Then data corresponding of all hybridizations (vector and labeled cDNA) were log_2_-transformed, normalized by the median of intensities by block then by array. Finally, signal processing was performed using vector oligonucleotide data to correct the relative amount of cDNA present in each spot. The microarray data from this research has been deposited in the NCBI Gene Expression Omnibus data repository under accession number GSE15614.

### Statistical analysis

Statistical analyzes were performed using the R software version 2.4.1 (R Development Core Team, 2006) and packages that can be downloaded either from the CRAN or the BioConductor site .

#### a) Search for differentially expressed transcripts

The search for differentially expressed transcripts had two purposes: first, find transcripts that clustered our data and second, find transcripts that were preferentially over-expressed in a particular condition (See flowchart, Additional File 7). Detection of these differentially expressed genes relies on statistical tests, typically t-tests, but these tests have in general low power for microarray analysis due to the lack of information on each individual gene [106]. A key and critical aspect of these tests is the modeling of the residual variances. To get the most information from our data, we decided to detect differentially expressed genes using tests based on variance modeling: the Limma for moderated F-tests based on a hierarchical Bayesian method [107] and the SMVar method for pairwise comparisons with moderated t-tests based on a structural model [31].

To determine differentially expressed transcripts between 12 samples (testis, *vas efferens*, nine epididymal zones, *vas deferens*) or between 10 samples (the nine epididymal zones and deferent duct), the moderated F-test available in the Limma R package was used [108]. To account for dependencies between the data from the same animal, an animal effect was included in the model. Then, in order to determine transcripts preferentially over-expressed in one particular condition (tissue), we searched iteratively for transcripts differentially expressed between 2 conditions with a moderated t-test of the SMVar R package [31]. To define a differentially expressed gene between 2 conditions, the difference of mean intensity between the 2 conditions and the variance were calculated to obtain a ratio of these 2 values (mean and variance). If this ratio is "high", the gene is considered as differentially expressed. SMVar improves the estimation of the variance for small number of replicates like in this study. This method is a "shrinkage" approach, that takes into account information from all the genes to estimate each individual gene statistic. This increases the sensitivity of the test since the numbers of parameters to estimate is decreased. Its originality resides in the fact that the program models one variance per condition and not directly the variance of the difference between the two conditions. Thus, it allows for heterogeneous variances between conditions. More explanation could be found in Jaffrezic et al, [31]. In both analyses, a Benjamini & Hochberg [109] correction was performed on the raw p-values to correct for multiple tests.

#### b) Clustering of tissue arrays and transcripts

Clustering is useful for organizing the genes and samples from a set of microarray experiments so as to reveal biologically interesting patterns. There are several possibilities to cluster microarray data; among them agglomerative (bottom-up) hierarchical clustering algorithms are the most commonly used. Hierarchical methods are especially useful, because they enable to simultaneously examine groupings of the data into many small clusters (e.g. repeat samples from the same tissue) and a few large clusters (e.g. different treatment groups). The use of a single hierarchical model ensures that groups at different levels are nested, facilitating interpretation. Bottom-up clustering, which successively joins objects, is good at identifying small clusters, but can provide sub-optimal performance for identifying some large clusters. Conversely, top-down methods are good at identifying a few large clusters, but weaker when there are many small clusters. Because we had no *a priori *about the size of clusters from our data we analyzed our data with one algorithm of each of these two methods of clustering.

We then defined an original combination of statistical methods using: (1) a hierarchical method (Hierarchical Ascendant Classification: HAC or Hierarchical Clustering: HCL) and (2) a partitioning method (Partitioning around medoid: PAM). The hierarchical method is an unsupervised method: the analysis of the data is done without any *a priori *on the number of classes expected, but a major weakness of the hierarchical algorithm is that an improper fusion of data at an early stage cannot be corrected later. In order to correct this weakness, at least partially, we decided to add a second clustering step with the partitioning method. We choose the PAM algorithm because, compared to the k-means approach, it: (a) accepts a dissimilarity matrix (missing values allowed), (b) is more robust as it minimizes a sum of dissimilarities instead of a sum of squared Euclidean distances, (c) provides a graphical display, the silhouette plot, which allows the user to select the optimal number of clusters [110]. The PAM algorithm is classically used as a supervised method, but we used it as a "mixed unsupervised" method by giving a range of possible numbers of classes instead of a fixed one. This way, the PAM defines itself the best number of classes that fit our data. Note that the objects (zones or transcripts) similarity metric was the 1 minus correlation-based distance. On the other hand, the criterion chosen to agglomerate two clusters was the Ward criterion that consists in merging two clusters with a minimum increases in the total. The tissues clustering, allowed us to obtain "transcriptomic units" defined as association of zones in which the most differential transcripts have the same expression profile. But this classification is not informative about transcripts. Therefore, we made transcripts clustering that found clusters of co-expressed transcripts. According to expression profile of transcripts in each cluster, it was possible to conclude that clusters contain transcripts preferentially expressed in some of the transcriptomic units defined previously but not in all.

#### Tissues clustering

To identify some transcriptomic units defined by the agglomeration of membrane arrays inside of which the majority of the studied transcripts have similar expression profiles, we used the list of differentially expressed transcripts obtained from the Limma analysis. To determine the number of classes for which our data are the best classified, we used the PAM from the Cluster R package [108]. The PAM gives a score for a defined number of classes. We also performed the classification with the hierarchical algorithm, the HAC, present in the same package and only the results common for both methods were kept.

#### Transcripts clustering

The aim of this analysis was to identify some characteristic evolution of gene regulation between the different tissues studied. This classification was performed similarly as the zone clustering, using the list of differentially expressed transcripts obtained with the Limma package.

### Sequence annotation

#### a) Nucleotidic information

Because pig genome is not yet entirely sequenced and assembled, in order to obtain the name of the gene coding for the cDNA clones spotted on the microarray and hence the maximum of physiological information, we have compared the complete (when available) or partial sequence (EST) of clones with the NCBI UniGene "unique" database (defined as the "longest, best" sequence from each UniGene cluster) of different mammalian species. cDNA sequences were compared to UniGene *Sus scrofa *(Build #33), *Homo sapiens *(Build #213), *Bos Taurus *(Build #92), *Mus musculus *(#173), *Rattus norvegicus *(Build #175) and *Canis familiaris *(Build #22) using BLASTN (v2.2.18) [111]. Two criteria depending on the database were used to parse and filter the blast results: For *Sus scrofa *database, we kept only sequences (subjects) with a global High-scoring Segment Pair (HSP) length of at least 200 nucleotides and 80% of identities with the cDNA sequence (query). For other databases, only sequences with a global HSP length of at least 200 nucleotides and 70% identities with the cDNA sequence were kept. For EST sequences with a length around 200 nucleotides that were still not annotated, we used the following criteria to filter their blast results: for *Sus scrofa *database, we kept only sequences with a minimum global coverage of 50% of the cDNA sequence with 80% of identities. For other databases, we kept only sequences with a minimum global coverage of 50% of the cDNA sequence with 70% of identities. Then for cDNA sequences that pass these filters, we kept the UniGene ACC corresponding to the sequence with the best score for each species to annotate the corresponding clone.

#### b) Protein information

The UniGene ACC were further used to obtain the corresponding UniProt ACC using the ID Mapping tool on the UniProt web site . When a UniGene ACC mapped to several UniProt ACC, we kept the one corresponding to the SwissProt databank. These UniProt ACC were used to annotate the corresponding clone.

#### c) Biological network and pathway analysis

Gene ontology annotations associated to the UniProt ACC were obtained on the Gene Ontology Annotation (GOA) Database .

#### Gene Ontology term enrichment

To have the maximum information, we worked with a multi-species approach. For each clone, we have kept the Gene Ontology information available for the UniProt ACC corresponding to the Homo UniGene ACC hit, otherwise we have kept the Gene Ontology information available for the UniProt ACC corresponding to the mouse UniGene ACC, then to the rat, then to the bovine, to the dog and at least the pig. The Gene Ontology term enrichment analysis was done with the data corresponding to transcripts clustered by classification analyses with 12 and 10 tissues for the 3 main GO terms independently (Biological process, Cellular component and Molecular function). For each cluster, we have retained a unique UniProt ACC (to eliminate multiple spotted genes). We have searched for Gene Ontology terms that were enriched in one cluster compare to all the clusters obtained by the classification analyses with 12 and 10 zones using EASE program [112]. A Benjamini & Hochberg [109] correction was performed on the raw p values to correct for multiple tests for each Gene Ontology term. A term was defined as significantly enriched when it had an adjusted p-value lower or equal to 15%.

### Validation of microarray Results by PCR and Q-PCR

#### a) PCR

The reverse transcriptase assay was performed on 3 μg total RNA using the Superscript Reverse transcriptase H (Invitrogen, Cergy Pontoise, France) and oligo(dT)_15 _primers. PCR was performed at the temperature specified for the primer sets (see Additional File 2) and a final elongation step at 72°C for 5 min. Aliquots of each reaction mixture were analyzed on a 2% ethidium bromide stained agarose gel. PCRs on tissues were performed for 25, 30, 35 and 40 cycles. *RPL19 *primers (Additional File 2) were used as PCR controls and to correct the quantity of mRNA after digital analysis of the signal. For all amplicons, sequencing was done at least once in order to validate the primers.

#### b) QPCR

For each set of primers (Additional File 8) a standard curve was constructed using a serial dilution of cDNA solution. These curves were generated using dilution coefficient versus the mean threshold cycle (Ct). The linear correlation coefficient (R2) ranged from 0.97 to 0.99. Based on the slopes of the standard curves, the amplification efficiencies of the standards ranged from 1.8~2.2, (derived from the formula E = EXP(-1/slope)). This calculation method results in efficiencies higher than 100%, which was an overestimate of the "real efficiency". Each PCR reaction also included a non-template negative control to check for primer-dimer. Each reaction consisted of 20 μL containing 3 μL of cDNA (diluted 5 times) and 9 pmol of each primer set. The real time qPCR was run on a iCycler system (Bio-Rad, Marnes-la-Coquette, France). The cycling conditions were 1 cycle of denaturation at 95°C/3 min, followed by 40 three-segment cycles of amplification (95°C/30 sec, 58°C/30 sec, 72°C/20 sec) where the fluorescence was automatically measured during elongation and melting steps (80 cycles of 30 sec with a temperature range from 55 to 96°C). The baseline adjustment method of the iCycler system software (Bio-Rad, version 3.1) was used to determine the Ct in each reaction. A melting curve was constructed for each primer pair to verify the absence of dimerization. All samples were amplified in duplicates and the mean was used for further analysis. To compare expression level of a gene between different tissues studied, the mean Ct of each sample was corrected by this obtained for *RPL19*.

### Comparison between mammalians transcriptomic data

A comparison of epididymal transcriptomic data obtained for mouse, rat, human and boar was realized. For mouse and rat, expression dataset were downloaded from the Mammalian Reproductive Genetics website  and annotation files were obtained from Affymetrix website. For mouse, we used the same criterion as in Johnston et al. [21] to consider qualified genes. Then, mouse UniGene IDs corresponding to Affymetrix IDs was obtained (this represents 14418 ID). For rat, we applied the criterion defined by Jelinsky et al. [20] to consider qualified genes. Then, we obtained the rat UniGene IDs corresponding to Affymetrix IDs (15072 IDs). These rat UniGene IDs were transformed in mouse homologous UniGene ID using NCBI E-Utilities. We then obtained 11008 homologous mouse Unigene IDs.

For human, the list of genes considered detectable in the epididymis from Thimon et al. [22] study was kindly provided to us by Dr Sullivan (Université Laval, Québec, Canada). Then, human UniGene IDs corresponding to Affymetrix IDs for qualified genes were obtained using annotation file downloaded from the Affymetrix website (34097 ID). As for rat, we have obtained corresponding mouse UniGene ID (final list: 23774 ID). For boar, we used transcripts that were differentially expressed between the 12 and 10 conditions for which we have been able to associate UniGene IDs (mouse, human, rat, bull, dog and boar). Using NCBI E-Utilities, we obtained 1550 homologous mouse UniGene ID.

## Authors' contributions

BG was involved in the experimental design and in planning the study, carried out sample collection, extracted RNA, carried out microarray molecular work, RT-PCR and real-time PCR work, performed statistical analyses, interpreted data and drafted the paper. GM and FJ developed and adapted the SMVar library, contributed to the statistical analyses of the microarray data and to the writing of the manuscript. MJM and SS contributed to the animal selection and fertility data processing. JLG and JLD conceived the study, coordinated the experimental design, contributed to the samples collection and to the microarray molecular work, to the analysis and interpretation of data, and to writing the paper. All authors have read and approved the final manuscript.

## Supplementary Material

Additional file 1 **List of potential markers for each of the 7 transcriptional units**. For each transcript defined as potential marker, the first column gives the transcriptional unit for which it was found to be a potential marker, the second gives its index on the array, columns 3 and 4 show the UniGene ACC and UniGene definition obtained by BLAST and column 5 shows its mean intensity measured for the transcriptional unit of interest. Click here for file

Additional file 2 **Gene-specific primers used for PCR study**. For each primer pair is given the Gene Symbol, the Gene name, sequences of forward and reverse primers, the length of amplified fragment, the GenBank Accession of the sequence used to design primers and the EMBL Accession of the sequenced amplified fragment. Click here for file

Additional file 3 **Transcripts corresponding to analysis of Gene Ontology term enrichment for clustering with 12 tissue samples**. For each GO term defined as enriched, the first column gives its definition, columns 2 and 3 show its GO ID and the class it belongs to (B_P: Biological Process, C_C: Cellular Component and M_F: Molecular Function). Column 4 gives the transcript cluster in which it was enriched. The last 5 columns show the index on the array, UniGene ACC, UniProtKB ACC, UniprotKB ID and UniprotKB definition of transcripts corresponding to GO terms enriched, respectively. Click here for file

Additional file 4 **Transcripts corresponding to analysis of Gene Ontology term enrichment for clustering with 10 tissue samples**. For each GO term defined as enriched, the first column gives its definition, columns 2 and 3 show its GO ID and the class it belongs to (B_P: Biological Process, C_C: Cellular Component and M_F: Molecular Function). Column 4 gives the transcript cluster in which it was enriched. The last 5 columns show the index on the array, UniGene ACC, UniProtKB ACC, UniprotKB ID and UniprotKB definitions of transcripts corresponding to GO terms enriched, respectively. Click here for file

Additional file 5 **Common over-expressed genes in the same epididymal unit between mammal species (Sus scrofa, Mus musculus, Rattus norvegicus and Homo sapiens)**. For each gene found over-expressed in the same epididymal unit between the 4 species, column 1 gives the unit for which it was found to be over-expressed. Column 2 shows its index on the pig array and the last 2 columns give its UniGene ACC and UniGene definition in mouse species. Click here for file

Additional file 6 **List of boar potential markers for which a knockout has a reproductive effect on mouse (MGI database)**. For each potential marker for which a knockout has a reproductive effect on the mouse, column 1 gives the transcriptional unit for which it is a potential marker. Column 2 shows the UniGene ACC corresponding to this marker used to obtain the Mouse.Ensembl.Gene.ID (column 3). Column 4 gives the MGI ACC associated with the marker and columns 5 and 6 show the symbol and the name of this marker, respectively. The last column gives a short description of the reproductive system phenotype. Click here for file

Additional file 7 **Statistical methods**. This flowchart summarizes our statistical methods in 3 steps. First (in blue), we search for differentially expressed transcripts between our 12 or 10 tissue samples in order to discover the classes of co-expressed transcripts and transcriptional units. Results of sample classification were used to search for over-expressed transcripts in a single transcriptional unit (orange). Finally, potential markers were transcripts which were common for one unit in cluster and over-expressed lists (purple). Click here for file

Additional file 8 **Gene-specific primers used for realtime PCR study**. For each primer pair is given the Gene Symbol, the Gene name, sequences of forward and reverse primers, the length of amplified fragment and the GenBank Accession of the sequence used to design primers. Click here for file
