# Supplementary material for: The adult boar testicular and epididymal transcriptomes
Source: BMC Genomics. 2009 Aug 7;10:369. doi: 10.1186/1471-2164-10-369 (PMC2738690; doi:10.1186/1471-2164-10-369)
Supplement: Additional file 2 — Gene-specific primers used for PCR study. For each primer pair is given the Gene Symbol, the Gene name, sequences of forward and reverse primers, the length of amplified fragment, the GenBank Accession of the sequence used to design primers and the EMBL Accession of the sequenced amplified fragment. [file 1471-2164-10-369-S2.pdf]

Gene symbol	Gene name	Primer sequence (5'⟶3')		Amplicon length	GenBank Accession	Amplicon Accession *
		Forward	Reverse			
RLP19	ribosomal protein L19	GGTACTGCCAATGCTCGAAT	CCATGAGAATCCGCTTGTTT	172	AF435591	
TSPAN1	tetraspanin 1	TTCCAGCGCCTCTAAGAGAA	CTGATTTGCACATGCTCAGG	161	AK231729	FM995255
METTL7B	methyltransferase like 7B	TTCCCCTACGTGATGGATGT	TTGTCAGGAACGTCTGAAAGTG	220	BX669720	FM995250
Ce9	CE9 protein	CTGGGCATGTTTCTGATCCT	GCCGGAGATTGGTCTGAATA	237	BX673259	FM995236
SLPI	secretory leukocyte peptidase inhibitor	CCCAATCACTGCAAGACAGA	GGTGCTGAGATGTTCTGCAA	242	NM_213870	
INSL3	insulin-like 3	AAATCAGACTCCCCTCAAACC	AGGGGTGTCATGAAATGCAG	127	NM_213970	
SPP1	secreted phosphoprotein 1	GCTGCAGACCAAGGAAAATC	TTCTGTGGCGCTAGGAAAGT	202	NM_214023	
CPE	carboxypeptidase E	AACCGGAACTTTCCTGACCT	AGGTTAGCCGAGAGCACAAA	179	NM_001097439	
Awn	sperm associated AWN protein	TCAGGTGCATGGAACAGAAG	AAGACTTATGCCTCCGCAAA	243	AJ853850	
APOD	apolipoprotein D	TCACCTGGATCATGTTTGGA	GGACGCAAAGCAAAAGAAAG	195	BX668247	
PTGDS	prostaglandin D2 synthase	AGCTGGTTCCTGGAGAAGAA	ATCAGAGTTCGGGTCACACA	128	NM_214228	
LCN2	lipocalin 2	GAGCAAGGCCGGTTTAAGAT	CGACATAGCTCTGCAATCCA	190	AK240091	FM995245
Lcn5	lipocalin 5	TCAACGCCACCCAGTTTT	GGCCATGACTTTCTCGTTGT	368	BX667222	FM995246
LCN8	lipocalin 8	CCAAAACCTGGTCCTGAAGA	TGGCGTATTGCTCGTAGTCA	212	BX922522	FM995248
LCN6	lipocalin 6	ACAGTGGAGCTGCTGAAACA	TGTGTGAACACCACAGCGTA	116	BX921778	FM995247
Ucal-P19	Uterocalin	ACCCCAACTTCAATGAAACG	TGGGTGCAGATGATAGCGTA	298	BX917960	FM995249
Unq2541	Uncharacterized lipocalin UNQ2541/PRO6093 precursor	ATGTTCACCAGCAACGTCAC	AGAGCAGGCATCTGACTTGG	296	BX919492	
WFDC3	WAP four-disulfide core domain 3	AGTTTGGCGATGAATGTCCT	CCAGGCAAGTCTCTGGAAGT	420	BX666624	FM995242
WFDC2	WAP four-disulfide core domain 2	GGCACAGGAGCAGAGAAAAG	CAGTTGCTGTCAGCTCAGGA	421	AJ506744	
WFDC10AL	WAP four-disulfide core domain 10A like	CGCCTTCAGATATCCACGAT	GCTGACAATATGACAGCTTAGGG	488	BX915529	FM995241
Defb19	defensin beta 19	AACCTGCTCCAAAGGACTCA	TCAAGGATGATGGATGGACA	246	BX921687	
DEFB109	defensin beta 109	GGTTTGGCTTCTGCTGAAAA	TTGGGACAGGCAAAAGAATC	145	BX671194	
DEFB113	defensin beta 113	CCAGCAGTTTTACGGAGGAA	TATTCCCAGGTGTTGCATGA	113	BX920299	FM995235
DEFB129	defensin beta 129	TCCGTGGGTGAATCTCTTCT	CAAATGGCACCACTGTGAAC	115	BX926011	
Eg627821	predicted gene, EG627821 (Alternate transcript 1)	ACTCAGTGGCAATCCCTTCA	TCAAGGAGCTCCATCTGGTT	212	BX924253	FM995231
Eg627821_Δ	predicted gene, EG627821 (Alternate transcript 2)	TAACCCAGAAGTGGACACCA	GAAAGCTTAAGAGGAGGTGTGG	289	BX922624	FM995232
CST11	cystatin 11 (Alternate transcript 1)	GTTGTTCCTCTCTGCCTTGG	GGTGGCCTTCAGTTACCAAA	247	BX676448	FM995238
CST11_Δ	cystatin 11 (Alternate transcript 2)	GTTGTTCCTCTCTGCCTTGG	GGTGGCCTTCAGTTACCAAA	169	BX676099	FM995239
Spink5l2	Kazal type serine protease inhibitor 5-like 2	CCAAATCCTTCCCAATCCTC	GCACAAGATGCAGGGGTTAT	236	BX924795	FM995240
MON1B	MON1 homolog B (yeast)	TACACCCCTGGGTGAAAAGA	TTACCAGGGTAGGGTTTGTCA	937	BX665264	
FXYD2	FXYD domain containing ion transport regulator 2	TATGAGACAGTCCGCAATGG	TAATAGGCCGGTGCTTCTTG	115	BX674298	
GP2	glycoprotein 2 (zymogen granule membrane)	AACCTGGTGCTGAGGAATTG	GACGGTTGGCACTGTTCTTT	245	BX676715	FM995243
MUC15	mucin 15, cell surface associated	AACCAGCTGCACTTCCTGTT	AAAGATCCGTGGCACATTCT	147	BX920827	FM995251
GLB1L3	galactosidase, beta 1-like 3	TGGGCCTCAGAAAACACTCT	AAATCCAGCAATCCAAGCAG	161	BX914549	FM995233
INHA	inhibin, alpha	CACCCTCCCAGTTTCATCTT	GCACCGTCTCGTACTTGAAA	223	NM_214189	
CLU	Clusterin	TTCTTTAACCGGGAGCCTTT	TGGTGGATCATGTCGAAGAA	176	NM_213971	
RARRES2	retinoic acid receptor responder (tazarotene induced) 2	CCTTTGTGAGGCTGGAATTT	TTGATGCAGGCTAGGCACTT	118	NM_001123186	
GPX5	glutathione peroxidase 5 (epididymal androgen-related protein)	GGTCTGACAGCCCAGTATCC	TCTTGCTTCCCAAATTGGTT	104	NM_213886	
HEXA	hexosaminidase A (alpha polypeptide)	ACATATGGCCCTGACTGGAA	TCAGCAGTTCACATCGGAAG	250	NM_001123221	
RAP1A	RAP1A, member of RAS oncogene family	AGTGCCAGCATTCCAGACTT	GGGAACTTGTGCAAACCAAT	103	BX920256	FM995252
CES7	carboxylesterase 7	TACTGTGCTGGGAAACGACA	TCTGGAGGCACAATTTAGGG	155	scac0031.h.09	FM995237
MAN2B2	mannosidase, alpha, class 2B, member 2	TAGAAGAGCCAAGGCAGACTTC	AATGTCCGGATTTCCTTCG	300	NM_213849	
MUC1	mucin 1, cell surface associated	TTCTGGCCTTCCAAGACAGT	ACAGTTCTTTCGTCGGCACT	257	AY243508	
CRABP1	cellular retinoic acid binding protein 1	GCCTTCACTCCCGAACATAA	CAGGACGGGGATCAATTCTA	283	BX674255	
RERG	RAS-like, estrogen-regulated, growth inhibitor	TGCCAGGCACAGTAAGAAGA	GGGGCCATAGTAGGGAAAAA	219	scac0031.i.20	FM995253
APLP2	amyloid beta (A4) precursor-like protein 2	TCATTGACGAGACCCTGGAT	TACTGCCTCTTCCGAAGCAT	202	BX924404	FM995230
B2M	beta-2-microglobulin	TGAAAAACGGGGAGAAGATG	GTGATGCCGGTTAGTGGTCT	189	L13854	

Gene symbol in capital are approved by the HUGO Gene Nomenclature Committee

* : Amplicons were sequenced and after identification were deposited at EBI with gene annotation.
