# Supplementary material for: The adult boar testicular and epididymal transcriptomes
Source: BMC Genomics. 2009 Aug 7;10:369. doi: 10.1186/1471-2164-10-369 (PMC2738690; doi:10.1186/1471-2164-10-369)

STATISTICAL METHODS

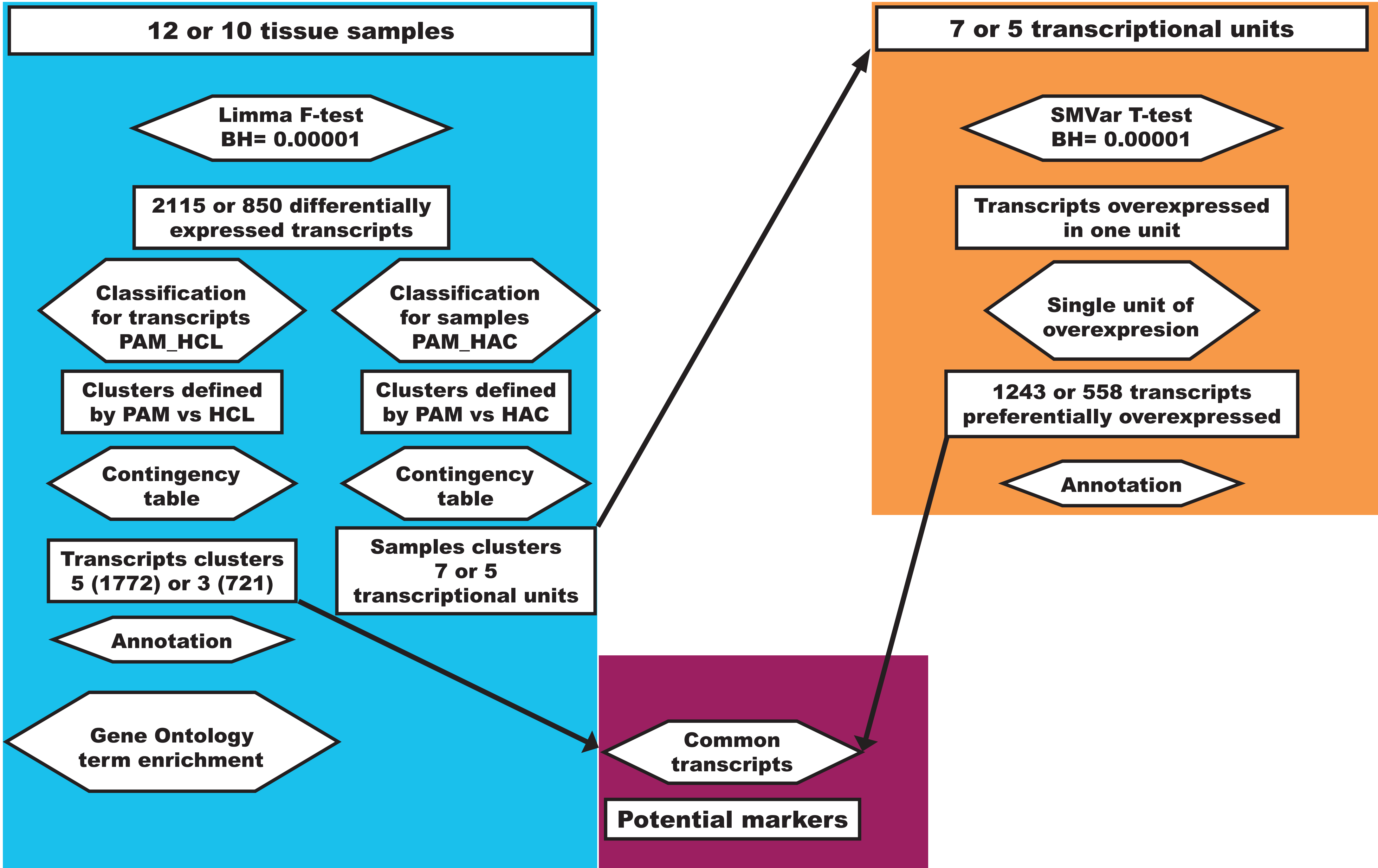

Supplement: Additional file 7 — Statistical methods. This flowchart summarizes our statistical methods in 3 steps. First (in blue), we search for differentially expressed transcripts between our 12 or 10 tissue samples in order to discover the classes of co-expressed transcripts and transcriptional units. Results of sample classification were used to search for over-expressed transcripts in a single transcriptional unit (orange). Finally, potential markers were transcripts which were common for one unit in cluster and over-expressed lists (purple). [file 1471-2164-10-369-S7.pdf]
