# Supplementary material for: The adult boar testicular and epididymal transcriptomes
Source: BMC Genomics. 2009 Aug 7;10:369. doi: 10.1186/1471-2164-10-369 (PMC2738690; doi:10.1186/1471-2164-10-369)
Supplement: Additional file 8 — Gene-specific primers used for realtime PCR study. For each primer pair is given the Gene Symbol, the Gene name, sequences of forward and reverse primers, the length of amplified fragment and the GenBank Accession of the sequence used to design primers. [file 1471-2164-10-369-S8.pdf]

Gene symbol	Gene name	Primer sequence (5'→3')		Amplicon length	GenBank Accession
		Forward	Reverse		
RLP19	ribosomal protein L19	ATCGCCAACGCCAACTCC	CCCTTCACTTTCAGGTACAGAC	293	AF435591
INSL3	insulin-like 3	TCTGGTGACCTCCTGATGCC	GATCCTTAGTGTCTGGTATTTCTGC	202	NM_213970
METTL7B	methyltransferase like 7B	ACGCCCTGGTCCGACTCC	TTGCTCTCTATCTTGCGGTTGTTC	157	AK232407
Ce9	CE9 protein	GACTTTATCACACACGACTCATCTC	CACATCTACACACCCACTCAGG	293	BX673259
SPP1	secreted phosphoprotein 1	CACGCTGACGACGCTGAC	GCTGGGCAACGAGGATGG	289	NM_214023
TSPAN1	tetraspanin 1	TGGTCGCCCTGGTCTACAC	TGAAGCCACAGCACTTGAGC	144	AK231729
CPE	carboxypeptidase E	GCTGTCATTCATTGGATTATGGATATTC	GCTGTCATCGTCGTTCTTGC	231	NM_001097439
Awn	sperm associated AWN protein	CTGTGTCTGGACGATCAAGGTG	TGGTGGCGATGTTGGAAGAAG	182	AJ853850

Gene symbol in capital are approved by the HUGO Gene Nomenclature Committee
